# A Complex-Valued Oscillatory Neural Network for Storage and Retrieval of Multidimensional Aperiodic Signals

**DOI:** 10.3389/fncom.2021.551111

**Published:** 2021-05-24

**Authors:** Dipayan Biswas, Sooryakiran Pallikkulath, V. Srinivasa Chakravarthy

**Affiliations:** ^1^Laboratory for Computational Neuroscience, Department of Biotechnology, Bhupat and Jyoti Mehta School of Biosciences, Indian Institute of Technology Madras, Chennai, India; ^2^Department of Mechanical Engineering, Indian Institute of Technology Madras, Chennai, India

**Keywords:** supercritical Hopf oscillator, power coupling, complex coupling, normalized phase difference, fourier decomposition, complex valued oscillator, complex Hebb’s rule, generative model

## Abstract

Recurrent neural networks with associative memory properties are typically based on fixed-point dynamics, which is fundamentally distinct from the oscillatory dynamics of the brain. There have been proposals for oscillatory associative memories, but here too, in the majority of cases, only binary patterns are stored as oscillatory states in the network. Oscillatory neural network models typically operate at a single/common frequency. At multiple frequencies, even a pair of oscillators with real coupling exhibits rich dynamics of Arnold tongues, not easily harnessed to achieve reliable memory storage and retrieval. Since real brain dynamics comprises of a wide range of spectral components, there is a need for oscillatory neural network models that operate at multiple frequencies. We propose an oscillatory neural network that can model multiple time series simultaneously by performing a Fourier-like decomposition of the signals. We show that these enhanced properties of a network of Hopf oscillators become possible by operating in the complex-variable domain. In this model, the single neural oscillator is modeled as a Hopf oscillator, with adaptive frequency and dynamics described over the complex domain. We propose a novel form of coupling, dubbed “power coupling,” between complex Hopf oscillators. With power coupling, expressed naturally only in the complex-variable domain, it is possible to achieve stable (normalized) phase relationships in a network of multifrequency oscillators. Network connections are trained either by Hebb-like learning or by delta rule, adapted to the complex domain. The network is capable of modeling N-channel electroencephalogram time series with high accuracy and shows the potential as an effective model of large-scale brain dynamics.

## Introduction

Currently, there are two prominent approaches to characterizing the neural code: the instantaneous rate or frequency at which the neuron fires action potential (“the spike frequency code” or the “rate code”) and the time of the occurrence of action potential (“spike time code”). The former assumes the information is processed/encoded over a larger time scale conveyed by average number of spikes fired in a given duration forms the basis of a large class of neural networks called the rate coded neural networks (Lippmann, 1990; Ruck et al., 1990; Lawrence et al., 1997). Whereas the latter believe that the precise timing of the action potential fired by a neuron encodes the ongoing activity, giving rise to a broad class of spiking neural network (Maass, 1997a, b; Izhikevich, 2003, 2004; Ghosh-dastidar and Lichtenstein, 2009). These two classes of neural networks are capable of universal function approximation and well explored (Maass, 1997a; Auer et al., 2008). They have been reported to solve a wide range of information processing problems like vector space transformation, dimensionality reduction, sequence processing, memory storage as an attractor and autoencoding (Hopfield, 1982; Frasconi et al., 1995; Kohonen, 1998; Trappenberg, 2003; Schmidhuber, 2015).

The third class of neural code, dubbed the oscillation coding (Fujii et al., 1996) also has been around for more than two decades. However, it has not been adopted in modeling literature as extensively as the first two types of neural code. Oscillation coding is based on the idea that not single-neuron activity, but the synchronized collective activity of a neural ensemble is the true building block of the brain’s activity. Unlike the rate code, which is often represented as a continuous signal encoding information as an average neural activity, or the spike time code, which is often described as a train of delta functions, oscillation code is a smooth signal that is said to be composed of distinct frequency bands. Oscillation coding also offers an opportunity to represent important temporal phenomena like temporal binding via transient synchrony (Wang and Terman, 1995, 1997; Shi et al., 2008) or rhythmic behaviors such as locomotion (Ermentrout and Kopell, 1989). There have been comprehensive and valorous attempts to describe all brain function in terms of the oscillatory activity of neural ensembles (Draguhn, 2004; Buzsáki et al., 2012). However, since existing literature does not commit to the exact size of a “neural ensemble,” activity measured at different scales goes by different names, including local field potentials (LFPs), electrocorticograms (ECoGs), electroencephalograms (EEGs), etc.

Since oscillations are ubiquitous in the brain, one would naturally expect nonlinear oscillator models to be used extensively to describe brain function. However, models of brain function often use sigmoidal neurons (to represent the rate code), spiking neuron models (to represent the spike time code), or more detailed conductance-based or biophysical models. In the field of neural signal processing, for example, a large body of literature depicting various deep neural network architectures such as recurrent neural network, convolutional neural network and deep belief network have been used for EEG signal processing and classification at various scales which found application in the following areas: brain-computer interface, epilepsy, cognitive and effective monitoring, etc. (Craik and He, 2019; Roy et al., 2019). Nonlinear oscillators are used exclusively for describing oscillatory phenomena like rhythmic movements or feature binding by synchronization. In an attempt to correct this bias, it would be a worthwhile modeling exercise to construct general networks of nonlinear neural oscillators that can be used to describe a wide range of brain functions.

The coupling strategies between a pair of neurons differ depending on the neuronal dynamics defined by the mathematical representation of the neural code. Biophysical neuron models employ biophysically detailed synapse models for coupling whereas the spiking neuron models employ elementary single exponential alpha function or double exponential function for coupling (Destexhe et al., 1994). Various two-variable oscillator models such as FitzHugh Nagumo model, Van der Pol, Wilson-Cowan, Kuramoto and Hopf oscillator principally employ what may be described as “real coupling strategy” (Campbell and Wang, 1994; Toral et al., 2003; Low et al., 2005; Izhikevich, 2007; Breakspear et al., 2010; Hoff et al., 2014; Sadilek and Thurner, 2015). In real coupling strategy, only the main variables (x_1_ and x_2_) of the two oscillators interact directly. Delayed real coupling strategies have also been explored for several of these oscillator models (Wirkus and Rand, 2002; Saha and Feudel, 2017). The averaged behavior of pulse-coupled oscillators has also been analyzed (Ermentrout and Kopell, 1991). On the contrary, in the complex coupling strategy (Hoppensteadt and Izhikevich, 2000), the two variables of the oscillator (x and y) are combined to form a complex variable (z = x + iy), and the complex state of one oscillator influences the complex state of the other. In this case, both x and y variables of one oscillator influence the x and y variables of the other.

In this paper, we describe a general trainable network of neural oscillators. For the oscillator model, we choose the simple Hopf oscillator. One reason behind the choice of the Hopf oscillator is the elegant form its description assumes in the complex variable domain. We show that it is important to operate in the complex domain in order to define some of the novel modeling features we introduce in this paper. Particularly, we introduce the concept of “power coupling” by which it is possible to achieve robust phase relationships among oscillators with very different intrinsic frequencies. The proposed network of oscillators can be trained to model multi-channel EEG, thereby demonstrating the potential to evolve into a large-scale brain model.

The outline of the paper is as follows. Section “Prior Work: Complex Coupling” begins with the definition of Hopf oscillator in the complex domain and show how to adapt the intrinsic frequency of the oscillator to that of a sinusoidal forcing signal. Section “A Pair of Hopf Oscillators With Complex Coupling” describes the dynamics of a pair of coupled Hopf oscillators and shows the advantages in coupling with a complex coupling constant. Section “Coupling Two Hopf Oscillators With Different Natural Frequencies” highlights the difficulties in coupling a pair of Hopf oscillators with very different intrinsic frequencies, and Section “Power Coupling” Between a Pair of Hopf Oscillators” shows how the difficulties can be overcome by adopting “power coupling.” Gathering all the modeling elements developed so far, Section “Adaptive Hopf Oscillator With Complex Input or Complex Adaptive Hopf Oscillator” describes a network of oscillators that can model multi-channel EEG. A discussion of the work was presented in the last section.

### A Network of Complex-Valued Oscillators With Complex Coupling and Power Coupling

The canonical model of Hopf oscillator without any external input is described as follows in complex state variable representation $\left(statevariable:z=re^{i\emptyset };;i=\sqrt{-1}\right)$, Cartesian coordinate representation (*state variables*: *x*, *y*) and polar coordinate representation (*state variables*: *r*, ∅) respectively:

Complex state variable representation:

$$\dot{z}=z\left(\alpha +i\omega +\beta_{1}{\left|z\right|}^{2}+\frac{\epsilon \beta_{2}{\left|z\right|}^{5}}{1-\epsilon {\left|z\right|}^{2}}\right)$$

Cartesian coordinate representation:

$$\dot{x}=x\left(\alpha +\beta_{1}r^{2}+\frac{\epsilon \beta_{2}r^{5}}{1-\epsilon r^{2}}\right)-\omega y$$

$$\dot{y}=y\left(\alpha +\beta_{1}r^{2}+\frac{\epsilon \beta_{2}r^{5}}{1-\epsilon r^{2}}\right)+\omega x$$

Polar coordinate representation:

$$\dot{r}=\alpha r+\beta_{1}r^{3}+\frac{\epsilon \beta_{2}r^{5}}{1-\epsilon r^{2}}$$

$$\dot{\emptyset }=\omega$$

Depending on the parameter values (α, β_1_, β_2_), the autonomous behavior of the oscillator principally falls into four regimes: critical Hopf regime (α = 0, β_1_ < 0, β_2_ = 0), supercritical Hopf regime (α > 0, β_1_ < 0, β_2_ = 0), supercritical double limit cycle regime (α < 0, β_1_ > 0, β_2_ < 0, *localmaxima* > 0) and subcritical double limit cycle regime (α < 0, β_1_ > 0, β_2_ < 0, *localmaxima* < 0) (Kim and Large, 2015). The critical Hopf regime has a stable fixed point at the origin and has the ability to show a stable resonating response to the complex sinusoidal input (*Fe*^*i*ω_0_*t*^). The supercritical Hopf regime has an unstable fixed point at the origin and a stable limit cycle at $r=\sqrt{\frac{\alpha }{\left|\beta_{1}\right|}}$. In the supercritical double limit cycle regime, the system exhibits two limit cycles, one of which is stable while the other being unstable. In the subcritical Hopf regime, the system has one stable fixed point at the origin. However, it has the ability to show stable oscillation under the influence of complex sinusoidal input whose frequency is not too different from that of the oscillator (ω−ω_0_ < *ϵ*). Throughout the rest of the paper, we will be using supercritical Hopf regime (with α = μ, β_1_ = 1, β_2_ = 0), which can be defined as follows:

Complex state variable representation:

$$\dot{z}=z\left(\mu +i\omega -{\left|z\right|}^{2}\right)$$

Cartesian coordinate representation:

$$\dot{x}=x\left(\mu -r^{2}\right)-\omega y$$

$$\dot{y}=y\left(\mu -r^{2}\right)+\omega x$$

Polar coordinate representation:

$$\dot{r}=\mu r-r^{3}$$

$$\dot{\emptyset }=\omega$$

We now present a series of results related to a single oscillator, a coupled pair, and a network of Hopf oscillators in the supercritical regime defined above.

### Prior Work: Complex Coupling

#### Single Oscillator With Adaptive Frequency

It has been previously shown that when a Hopf oscillator is influenced by a real sinusoidal input signal, it can adapt its natural frequency to the frequency of the input signal if it follows the following dynamics (Righetti et al., 2005).

(1a) $$\dot{z}=z\left(\mu +i\omega -{\left|z\right|}^{2}\right)+\varepsilon I_{ext}\left(t\right)$$

(1b) $$\dot{\omega }=-\varepsilon I_{ext}\left(t\right)\sin\emptyset$$

Given, *z* = *re*^∅^ = *x* + *iy*. It can be recognized that the Hopf oscillator is perturbed by a real input signal *I*_*ext*_(*t*) with coupling strength ε. At steady-state *r* reaches $\sqrt{\mu }$ for ε = 0. If *I*_*ext*_(*t*) = *I*_0_*sin*⁡(ω_0_*t* + φ) (*I*_0_, ω_0_, and φ are the magnitude, frequency and phase offset of the sinusoidal input signal), the natural frequency, ω, adapts to the frequency ω_0_.

#### Adaptive Hopf Oscillator With Complex Input or Complex Adaptive Hopf Oscillator

When a Hopf oscillator is influenced by a complex sinusoidal input signal, the natural frequency of the oscillator can adapt to the frequency of input if the natural frequency of the oscillator is updated according to the following equation:

(2a) $$\begin{array}{c} \dot{z}=z\left(\mu +i\omega -{\left|z\right|}^{2}\right)+\varepsilon I_{ext}\left(t\right)\\ \;\;I_{ext}\left(t\right)=I_{0}e^{i\left(\omega_{0}t+\varphi \right)} \end{array}$$

(2b) $$\dot{\omega }=-\varepsilon \left(real\left(I_{ext}\left(t\right)\right)\sin\emptyset -img\left(I_{ext}\left(t\right)\right)\cos\emptyset \right)$$

(2c) $$\dot{\omega }=-\varepsilon I_{0}\sin\left(\emptyset -\omega_{0}t-\varphi \right)$$

In this scenario, *I*_*ext*_(*t*) is a complex sinusoidal signal. It is straight forward to derive the learning rule for the natural frequency of the oscillator if eq. 2a is represented in the Cartesian and polar coordinate forms, respectively, as follows:

(2a1) $$\begin{array}{c} \dot{x}=\left(\mu -r^{2}\right)x-\omega y+\varepsilon I_{0}\cos\left(\omega_{0}t+\varphi \right)\\ \dot{y}=\left(\mu -r^{2}\right)y+\omega x+\varepsilon I_{0}\sin\left(\omega_{0}t+\varphi \right) \end{array}$$

(2a2) $$\begin{array}{c} \dot{r}=\left(\mu -r^{2}\right)r+\varepsilon I_{0}\cos\left(\omega_{0}t+\varphi -\emptyset \right)\\ \dot{\emptyset }=\omega +\frac{\varepsilon I_{0}}{r}\sin\left(\omega_{0}t+\varphi -\emptyset \right) \end{array}$$

**FIGURE 1:**
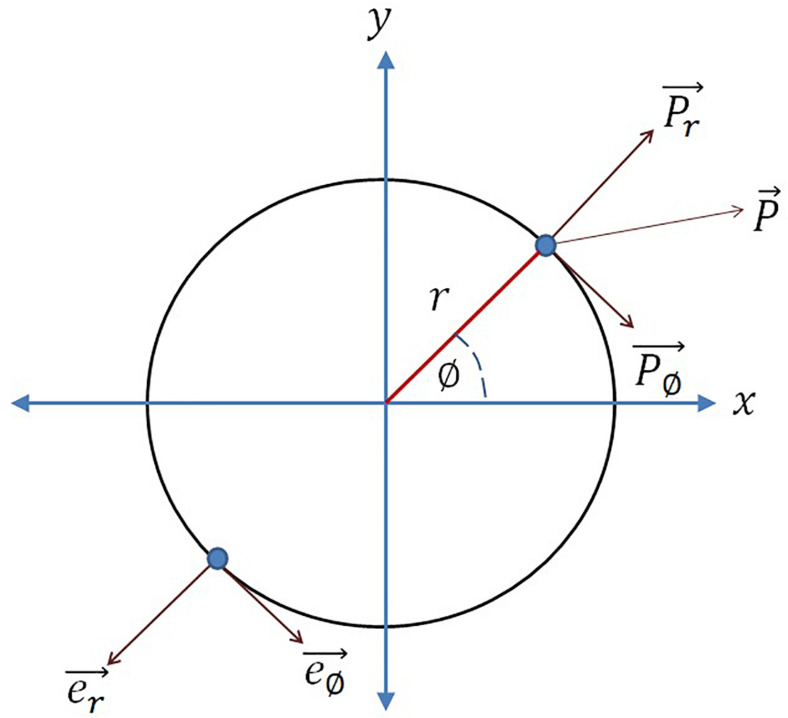
Considering the circle as the limit cycle of the Hopf oscillator, *e*_∅_ is the unit vector along increasing azimuth angle (∅), *e*_*r*_ is the unit vector along radius (*r*), *P* is the overall external input perturbation, *P*_∅_ and *P*_*r*_ are the external input perturbation along *e*_∅_ and *e*_*r*_ respectively. The analogy is drawn from Righetti et al. (2006).

In the phase plane representation, it can be observed from eq. 2a2 that the influence caused by the input perturbation on the oscillator phase is $\frac{\varepsilon I_{0}}{r}\sin\left(\omega_{0}t-\emptyset \right)$. Whereas from the Cartesian coordinate system representation (eq. 2a1) it can be observed that the overall influence of the external perturbation on the phase point along the tangential axis of the limit cycle ($\vec{e_{\emptyset }}$), $\vec{P_{\emptyset }}$ is (to understand the vector notations refer to Figure 1):

$$\begin{array}{c} r\vec{P_{\emptyset }}=real\left(\varepsilon I_{ext}\left(t\right)\right)\sin\emptyset -img\left(\varepsilon I_{ext}\left(t\right)\right)\cos\emptyset \\ =\varepsilon I_{0}\cos\left(\omega_{0}t+\varphi \right)\sin\emptyset -\varepsilon I_{0}\sin\left(\omega_{0}t+\varphi \right)\cos\emptyset \\ \vec{P_{\emptyset }}=\frac{\varepsilon I_{0}}{r}\sin\left(\emptyset -\omega_{0}t-\varphi \right) \end{array}$$

**FIGURE 2:**
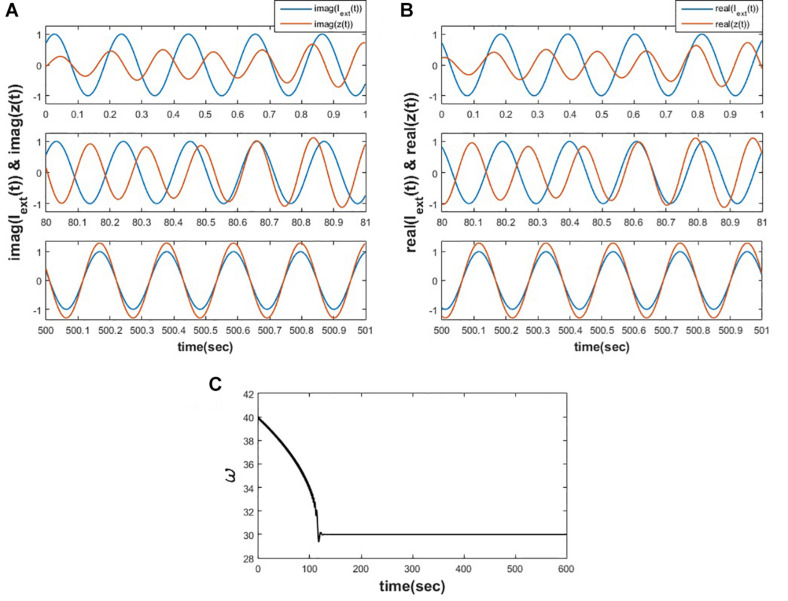
Equations 2a-c is simulated for $\mu =1,\omega \left(0\right)=40,\omega_{0}=30,\varepsilon =0.9,I_{0}=1,\varphi =\frac{\pi }{4},dt=0.001sec$ for 1000 s. **(A)** Depicts the variation of *y*(*t*) w.r.t *img*(*I*_*ext*_(*t*)) at various time instants. **(B)** Depicts the variation of *x*(*t*) w.r.t *real*(*I*_*ext*_(*t*)) at various time instants. **(C)** It can be observed that the natural frequency of the Hopf oscillator adapts to the frequency of the input signal.

This motivates us to adapt the learning rule for the natural frequency of the oscillator, as proposed in eq. 2c, by dropping the magnitude of oscillation because of the same reason as described by Righetti et al. (2006). We have simulated the eqs. 2a-2c and observed that the proposed learning rule for the natural frequency of the oscillator allows it to adapt to the frequency of the input complex sinusoidal signal, as shown in the following Figure 2.

#### A Pair of Hopf Oscillators Coupled Through Real Coupling

When two Hopf oscillators with equal natural frequencies are coupled with the real coupling coefficient as described below (eq. 3), they are going to phase lock in phase (0) or out of phase by (2*n* + 1)π depending on the polarity of the coupling and initialization. (for proof refer to Supplementary Appendix 1)

(3a) $$\dot{z_{1}}=z_{1}\left(\mu +i\omega_{1}-{\left|z_{1}\right|}^{2}\right)+W_{12}real\left(z_{2}\right)$$

(3b) $$\dot{z_{2}}=z_{2}\left(\mu +i\omega_{2}-{\left|z_{2}\right|}^{2}\right)+W_{21}real\left(z_{1}\right)$$

where *W*_12_ is the real coupling coefficient from 2nd oscillator to 1st one, and *W*_21_ is the real coupling coefficient from 1st to 2nd. In the stated scenario, both of the oscillators have identical natural frequencies, ω_1_ = ω_2_. This, with real-valued coupling, a pair of Hopf oscillators with equal intrinsic frequencies can only produce two possible values of phase difference.

#### A Pair of Hopf Oscillators With Complex Coupling

When two Hopf oscillators with identical natural frequencies are coupled bilaterally through complex coefficients with Hermitian symmetry, they can exhibit phase-locked oscillation at a particular angle similar to the angle of complex coupling coefficient. (for proof refer to the Supplementary Appendix 2).

(4a) $$\dot{z_{1}}=z_{1}\left(\mu +i\omega -{\left|z_{1}\right|}^{2}\right)+Wz_{2}$$

(4b) $$\dot{z_{2}}=z_{2}\left(\mu +i\omega -{\left|z_{2}\right|}^{2}\right)+W^{*}z_{1}$$

where *z*_1_ = *r*_1_*e*^*i*∅_1_^, *z*_2_ = *r*_2_*e*^*i*∅_2_^ and *W* = *Ae*^*i*θ^, *W*^∗^=*Ae*^−*i*θ^ to be the coupling coefficient (*A* and θ being the magnitude and the angle of complex coupling coefficient), in polar coordinate system representation:

(4a1) r1.=(μ-r12)r1+Ar2cos⁡(∅2-∅1+θ)∅1.=ω+Ar2r1sin⁡(∅2-∅1+θ)

(4b1) r2.=(μ-r22)r2+Ar1cos⁡(∅1-∅2-θ)∅2.=ω+Ar1r2sin⁡(∅1-∅2-θ)

At steady-state ∅_1_−∅_2_ approaches any of the solutions 2*n*π + θ depending on the initial conditions [∅_1_(0) and ∅_2_(0)], whereas the magnitude of the complex coupling coefficient determines the rate at which the phase-locking occurs. ψ_*ss*_ (where, ψ = ∅_1_−∅_2_ and ψ_*ss*_ is the steady state value of ψ) attains solution 2*n*π + θ for the following initial condition 2*n*π−(−π + θ) < ∅_1_(0)−∅_2_(0) < 2*n*π−(π + θ).

#### Simulated Result

**FIGURE 3:**
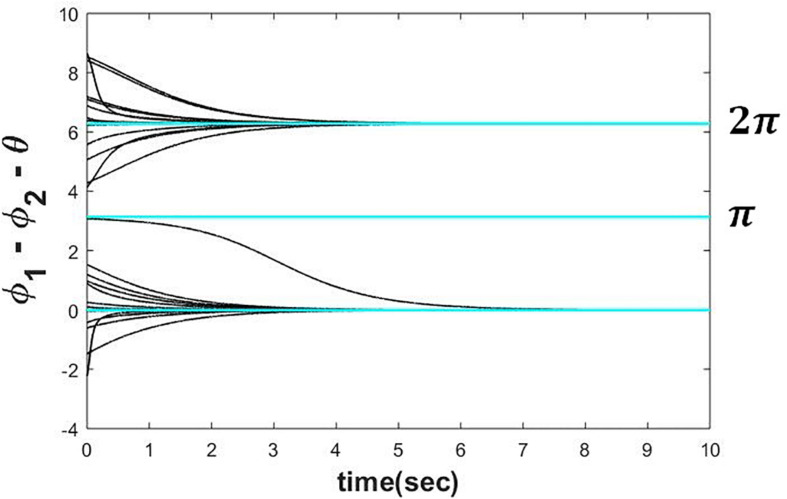
Two Hopf oscillators, dynamics of which is defined in the eqs. 4a1 and 4b1 simulated with ω = 5, μ = 1, *A* = 0.5, $\theta =\frac{\pi }{4}$ for various initial conditions (−π < ∅_1_(0)−∅_2_(0)−θ≤3π) depicting that ψss(=∅1ss-∅2ss) can reach any of the following solutions 2*n*π + θ depending on the initial condition.

To verify the above result, we have simulated eqs. 4a1 and 4b1 numerically by Euler integration method on MATLAB platform with △*t* = 1*msec*. It can be observed from the plot (refer to the Figure 3) that the steady-state phase difference between the two oscillators (ψ_*ss*_) is either achieving θ or 2π + θ depending on the whether it was initialized in the intervals θ − π < ψ(0) ≤ θ + π and θ + π < ψ(0) ≤ 2π + θ respectively.

#### Training Rule for the Complex Coupling

When a pair of complex sinusoidal inputs with identical frequency is presented to a pair of coupled Hopf oscillators with complex coupling and identical natural frequencies (eq. 4), and when the complex coupling coefficient is adapted according to eq. 5, the angle of the coupling coefficient approaches the phase difference of the external inputs. In other words, the complex coupling learns the phase relationship of the external inputs.

To train the complex coupling coefficient, a Hebbian like learning rule can be used as follows:

(5) τWW.=-W+z1z2*

The polar coordinate representation:

(5a) $$\tau_{W}\dot{A}=-A+r_{1}r_{2}\cos\left(\emptyset_{1}-\emptyset_{2}-\theta \right)$$

(5b) $$\tau_{W}\dot{\theta }=\frac{r_{1}r_{2}}{A}\sin\left(\emptyset_{1}-\emptyset_{2}-\theta \right)$$

We have limited the scope of our study only to θ dynamics by assuming $\dot{A}=0$. Assuming τ_*W*_ is the time constant for the specified learning dynamics. A similar adaptation of the Hebb’s rule was used earlier to train connections in a complex version of the Hopfield network (Hopfield, 1982). When the dynamics of two Hopf oscillators is defined by eq. 4 (with no external input), and the complex coupling coefficient is trained according to eq. 5 with very small value of *A*(|*W*|), θ(*angle*(*W*)) learns ∅_1_(0)−∅_2_(0). From eq. 4, it can be interpreted that ∅_1_(*t*) ≈ ω*t* + ∅_1_(0) and ∅_2_(*t*) ≈ ω*t* + ∅_2_(0)(*Assuming A*≪1), similarly from eq. 5, we can see that θ *or angle*(*W*) learns angle(z1z2*) or ∅_1_(*t*)−∅_2_(*t*) ≈ ∅_1_(0)−∅_2_(0). It is shown in the Supplementary Appendix 3 that the nonzero symmetric steady state solution (*r*_1*ss*_ = *r*_2*ss*_ ≠ 0, θ−ψ_*ss*_) is $r_{1ss}=\sqrt{\mu +A}$, ψ = 2*n*π + θ, which is a stable node.

On the other hand, in a network of two coupled oscillators driven by separate external sinusoidal forcing as described in the following eq. 6 with the same frequency as the natural frequency of the two oscillators but any phase offset (φ_1_, φ_2_) will drive those oscillators to oscillate at the same phase as the external sinusoidal forcing (∅_1_ ≈ ωt + φ_1_ *and* ∅_2_ ≈ ωt + φ_2_), provided very low magnitude of the complex coupling coefficient (*A*≪1).

(6) $$\begin{array}{c} \dot{z_{1}}=z_{1}\left(\mu +i\omega -{\left|z_{1}\right|}^{2}\right)+Ae^{i\theta }z_{2}+I_{01}e^{i\left(\omega t+\varphi_{1}\right)}\\ \dot{z_{2}}=z_{2}\left(\mu +i\omega -{\left|z_{2}\right|}^{2}\right)+Ae^{-i\theta }z_{1}+I_{02}e^{i\left(\omega t+\varphi_{2}\right)} \end{array}$$

Under the influence of external input, the angles of *z*_1_ and *z*_2_ (∅_1_ and ∅_2_ respectively) tend to ω*t* + φ_1_ and ω*t* + φ_2_, respectively. When the complex coupling coefficient is trained according to eq. 5, the *angle*(*W*) or θ learns angle(z1z2*) or ∅_1_(*t*)−∅_2_(*t*) ≈ φ_1_−φ_2_. During training, A must be kept low (A≪1) so that z_1_ and z_2_ dynamics is influenced only by the intrinsic oscillatory dynamics and the forcing signal. During testing, however, the external input is removed, A is again increased so that the phase difference between the oscillators is stable and determined by the coupling constant.

#### Simulated Result

**FIGURE 4:**
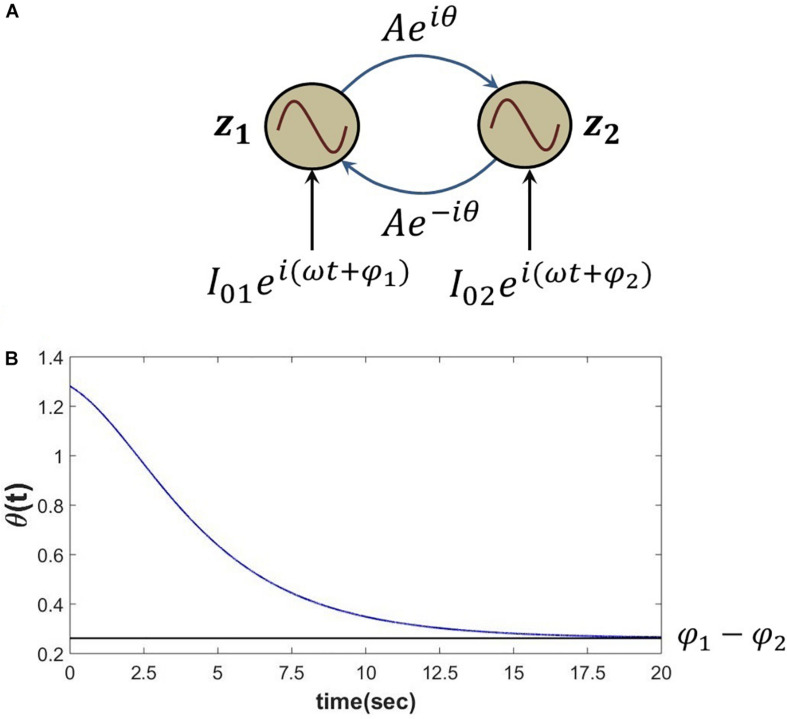
**(A)** Network schematic of dynamics represented in eq. 6 where the complex coupling coefficient is trained according to eq. 5. **(B)** It can be verified that for the following set of network parameters θ learns φ_1_−φ_2_. *I*_01_ = *I*_02_ = 0.3, $\varphi_{1}=\frac{\pi }{4},\varphi_{2}=\frac{\pi }{6},\omega =5,A=10^{-5},\eta =\frac{1}{dt},dt=0.001$.

The proposed Hebb like learning rule (eq. 5) allows the angle of complex coupling weight (θ) to learn the phase difference between the complex sinusoidal inputs driving each of the oscillators for very low magnitude of the complex coupling weight (*A*≪1). To verify this, eq. 6 is simulated for the set of parameters as described in the following Figure 4. It can be observed that θ learns the phase difference between the complex sinusoidal input signals (φ1-φ)2.

### Novel Contributions: Power Coupling

#### Coupling Two Hopf Oscillators With Different Natural Frequencies

A different kind of dynamics can be observed when two sinusoidal oscillators with different natural frequencies are coupled with real coupling coefficient. A pair of Hopf oscillators with real coupling shows Arnold tongue behavior which may be described as follows: the two oscillators entrain to commensurate frequencies (having a simple integral ratio) for specific ranges of the coupling strength, and the range of frequencies for which the entrainment occurs widens with the strength of the coupling coefficient (Izhikevich, 2007). There are whole ranges of the coupling strength and the natural frequencies where there is no specific phase relationship at all. Therefore, it is not easy to get a stable phase relationship between two Hopf oscillators with very different frequencies and real coupling.

The situation is a bit more facile with Kuramoto oscillators (Izhikevich, 2007). When a pair of Kuramoto oscillators are coupled according to the equation shown below, their natural frequencies converge to a particular frequency in between their natural frequencies, depending on the coupling strength.

$${\dot{\emptyset }}_{1}=\omega_{1}+K_{1}\sin\left(\emptyset_{2}-\emptyset_{1}\right)$$

$${\dot{\emptyset }}_{2}=\omega_{2}+K_{2}\sin\left(\emptyset_{1}-\emptyset_{2}\right)$$

Let, *K*_1_ and *K*_2_ be the real coupling strengths in the forward and backward directions, respectively. The phase difference between the two oscillators (∅ = ∅_1_ − ∅_2_) reaches a steady-state value if *K*_1_ + *K*_2_ > |ω_1_ − ω_2_| which is satisfied by the relationship sin⁡∅^∗^=$\frac{\omega_{1}-\omega_{2}}{K_{1}+K_{2}}$ where ∅^∗^ be the steady-state phase difference. Notably, both of the oscillators reach a periodic solution with frequency ω^∗^=$\frac{K_{1}\omega_{2}+K_{2}\omega_{1}}{K_{1}+K_{2}}$. In this scenario, although synchronization at a particular intermediate frequency with a particular phase difference is ensured, the natural frequency of oscillation is not maintained, i.e., to phase lock at a particular angle, the oscillation of both the oscillators had to converge at a particular intermediate value. A network of such Kuramoto oscillators (eq. 7a) with natural frequencies drawn from a Gaussian distribution with constrained standard deviation can show increased phase synchrony among the oscillators when the isotopic coupling strength (K) exceeds a threshold (Breakspear et al., 2010).

(7a) $${\dot{\emptyset }}_{i}=\omega_{i}+\frac{K}{N}\sum_{j=1}^{N}\sin\left(\emptyset_{j}-\emptyset_{i}\right)$$

$$\;\;re^{i\theta }=\frac{1}{N}\sum_{j=1}^{N}e^{i\emptyset_{j}}$$

Thus, we can see that the dynamics of a pair of coupled Hopf oscillators become dramatically more complicated when we relax the equality relationship between the natural frequencies of the coupled oscillators. We can visualize a simple, desirable extension of the dynamics of a pair of coupled oscillators with distinct natural frequencies. When the natural frequencies are equal, we found that the phase difference equals the angle of the complex coupling factor. However, when the natural frequencies are unequal, there cannot be a phase difference in the usual sense (∅_1_ − ∅_2_), although when the coupled oscillators are entrained in the *m*:*n* ratio, the phase difference has been defined as *m*∅_1_ − *n*∅_2_ (where, *m*, *n* are integers). Therefore, one may define a quantity called “normalized phase difference” as follows:

(7b) $$\psi_{12}=-\psi_{21}=\frac{\emptyset_{1}}{\omega_{1}}-\frac{\emptyset_{2}}{\omega_{2}}$$

Let ψ_12_ and ψ_21_ be the normalized phase difference of 1st oscillator w.r.t 2nd oscillator and vice versa. Is it possible to relate the normalized phase difference with the angle of the complex coupling coefficient? It turns out that such a relationship is not possible even with the complex coupling of eq. 4. To this end, we extend the complex coupling to a new form of coupling we label “power coupling” as described below.

#### “Power Coupling” Between a Pair of Hopf Oscillators

A pair of sinusoidal oscillators (Hopf or Kuramoto oscillator) can entrain at a specific normalized phase difference if they are coupled through complex “power coupling” coefficient according to the following eqs. 8a and 8b at a particular value dependent on the angle of complex “power coupling” coefficient, natural frequencies of the coupled oscillators and the initial values of the phase angle of those oscillators (for proof please refer to Supplementary Appendix 4).

Coupling a pair of Hopf oscillators through power coupling:

(8a) z1.=z1(μ+iω1-|z1|2)+A12eiθ12ω2z2ω1ω2

(8b) z2.=z2(μ+iω2-|z2|2)+A21eiθ21ω1z1ω2ω1

where, $A_{12}e^{i\frac{\theta_{12}}{\omega_{2}}}=W_{12}$ is the weight of power coupling from 2nd oscillator to the 1st oscillator and $A_{21}e^{i\frac{\theta_{21}}{\omega_{1}}}=W_{21}$ is the weight of power coupling from 1st oscillator to the 2nd oscillator.

The polar coordinate representation:

r1.=(μ-r12)r1+A12r2ω1ω2cos⁡ω1(∅2ω2-∅1ω1+θ12ω1ω2) (8a1)

∅1.=ω1+A12r2ω1ω2r1sin⁡ω1(∅2ω2-∅1ω1+θ12ω1ω2)     (8a2)

r2.=(μ-r22)r2+A21r1ω2ω1cos⁡ω2(∅1ω1-∅2ω2+θ21ω1ω2)(8b1)

∅2.=ω2+A21r1ω2ω1r2sin⁡ω2(∅1ω1-∅2ω2+θ21ω1ω2)     (8b2)

**FIGURE 5:**
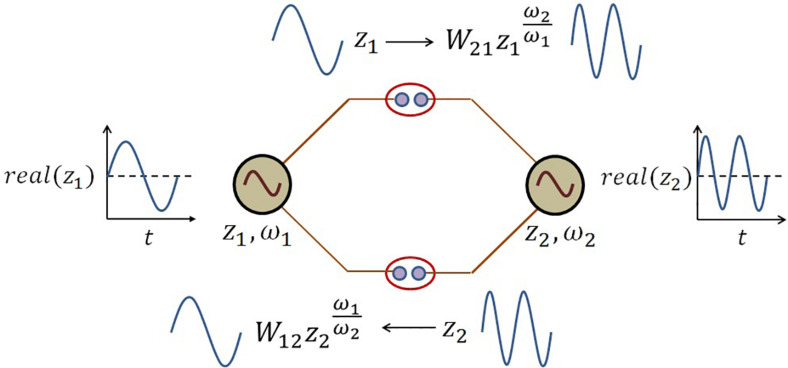
The schematic network representation of eq. 8. There is a kind of frequency transformation occurring at the *power coupling* synapse because of the following term in the dynamical eq. 8:$\frac{\omega_{j}}{\omega_{i}}$. At the *power coupling* synapse, this frequency-transformed version of the oscillation from *j*^*th*^ oscillator is weighted by the power coupling weight $W_{ij}=Ae^{i\frac{\theta_{ij}}{\omega_{j}}}$ to convert the forcing signal from *j*^*th*^ oscillator to *i*^*th*^ oscillator and vice versa.

The schematic of the coupling architecture is elaborated in the following Figure 5.

Based on eqs. 8a2 and 8b2, we understand that two modified Kuramoto oscillators may be coupled using power coupling as follows:

(9) $$\begin{array}{c} \dot{\emptyset_{1}}=\omega_{1}+A_{12}\sin\omega_{1}\left(\frac{\emptyset_{2}}{\omega_{2}}-\frac{\emptyset_{1}}{\omega_{1}}+\frac{\theta_{12}}{\omega_{1}\omega_{2}}\right)\\ \dot{\emptyset_{2}}=\omega_{2}+{\text{A}}_{21}\sin\omega_{2}\left(\frac{\emptyset_{1}}{\omega_{1}}-\frac{\emptyset_{2}}{\omega_{2}}+\frac{\theta_{21}}{\omega_{1}\omega_{2}}\right) \end{array}$$

#### Power Coupling Among N Hopf Oscillators

Similarly, the dynamics of the *i*^*th*^ oscillator in a network of *N* supercritical Hopf oscillators coupled through *power coupling* can be represented as:

(10) zi.=zi(μ+iωi-|zi|2)+∑j=1∋j≠iNAijeiθijωjzjωiωj

Let $W_{ij}=A_{ij}e^{i\frac{\theta_{ij}}{\omega_{j}}}$ be the weight of power coupling from the *j*^*th*^ oscillator to *i*^*th*^ oscillator.

The polar coordinate representation is:

(11a) ri.=(μ-ri2)ri+∑j=1∋j≠iNAijrjωiωjcos⁡ωi(∅jωj-∅iωi+θijωiωj)

(11b) ∅i.=ωi+∑j=1∋j≠iNAijrjωiωjrisin⁡ωi(∅jωj-∅iωi+θijωiωj)

Therefore, the dynamics of *the i*^*th*^ oscillator in a network of *N* number Kuramoto oscillators coupled through power coupling can be represented as:

(12) $$\dot{\emptyset_{i}}=\omega_{i}+\sum_{j=1∋j\neq i}^{N}A_{ij}\sin\omega_{i}\left(\frac{\emptyset_{j}}{\omega_{j}}-\frac{\emptyset_{i}}{\omega_{i}}+\frac{\theta_{ij}}{\omega_{i}\omega_{j}}\right)$$

We may appreciate the difference in the second term in eqs. 7a and 12. ψij.s′ (defined in eq. 7b) can be obtained from eqs. 8a2, 8b2 and 11b for a network of two oscillators and also for the general case of *N* (> 2) Hopf oscillators respectively:

(13a) ψ12.=A12ω1r2ω1ω2r1sin⁡ω1(∅2ω2-∅1ω1+θ12ω1ω2)-A21ω2r1ω2ω1r2sin⁡ω2(∅1ω1-∅2ω2+θ21ω1ω2)

(13b) ψij.=∑k=1∋k≠iNAikωkrkωiωkrisin⁡ωi(∅kωk-∅iωi+θikωiωk)-∑k=1∋k≠jNAjkωkrkωjωkrjsin⁡ωj(∅kωk-∅jωj+θjkωjωk)

At steady state, ψ_12_ and $\psi_{ij}\;\left(=\frac{\emptyset_{i}}{\omega_{i}}-\frac{\emptyset_{j}}{\omega_{j}}\right)$ can attain any of the possible solutions of the above eqs. 13a,b.

For a network of any arbitrary *N* number of Hopf oscillators as described in eqs. 10 and 11 under the constraint *e*^*i*θ_*ij*_^ = *e*^−*i*θ_*ji*_^ or θ_*ij*_ = −θ_*ji*_, *A*_*ij*_ = *A*,μ = 1, $\psi_{ij}^{\prime }$s can achieve the following desirable solution:

(13b.s) ψij*=θijωiωj

for certain initial conditions.

However, there can exist other solutions for which the sum of the terms on the right-hand side of eqs. 13a,b is zero, without all the individual terms being zero. That is eq. 13b.s above is not satisfied. We call these solutions spurious solutions.

Whether the final solution is a desirable or a spurious solution depends on the initial conditions. Therefore, the solution ψij* can be achieved only for certain initial values of $\emptyset {}_{i}^{\prime }$s.

#### Two Hopf Oscillators With Power Coupling

Under the following constraints θ_12_ = θ, θ_21_ = −θ, *A*_12_ = *A*_21_ = *A*, μ = 1, some of the steady-state solutions of eq. 13a where both entities on the right-hand side of eq. 13a is zero, is as follows:

ψ12ss=θω1ω2±n1πω1=θω1ω2±n2πω2

following the specification: $\frac{n_{1}}{n_{2}}=\frac{\omega_{1}}{\omega_{2}}$. It can be noticed that *n*_1_ = *n*_2_ = 0 gives us the desired solution, as mentioned in eq. 13b.s.

Let $\sigma_{12}=\psi_{12}-\frac{\theta }{\omega_{1}\omega_{2}}=\frac{\emptyset_{1}}{\omega_{1}}-\frac{\emptyset_{2}}{\omega_{2}}-\frac{\theta }{\omega_{1}\omega_{2}}$, so, $\sigma_{ij}=\frac{\emptyset_{i}}{\omega_{i}}-\frac{\emptyset_{j}}{\omega_{j}}-\frac{\theta_{ij}}{\omega_{i}\omega {}_{j}}$.

**FIGURE 6:**
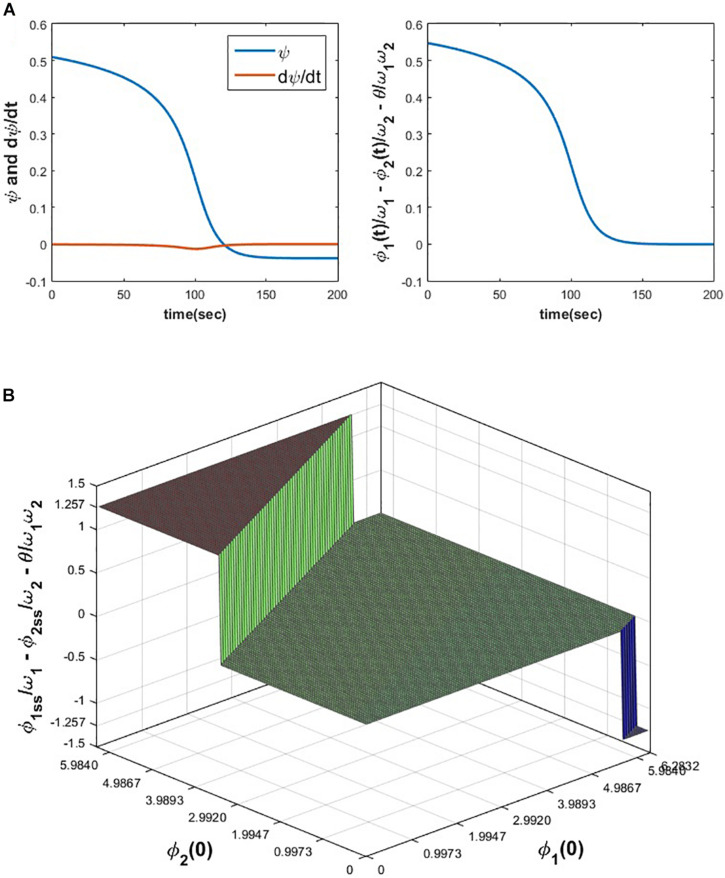
**(A)** The variation of ψ, $\dot{\psi }$, and $\psi -\frac{\theta }{\omega_{1}\omega_{2}}$ w.r.t time is plotted by simulating eqs. 8a1 and 8b1 for the following set of parameters and initial conditions. ω_1_ = 5, ω_2_ = 10, *A* = 0.05, θ = −1.8968, ∅_1_(0) = 3.7008, ∅_2_(0) = 2.3106. **(B)** It can be verified that depending on the various combinations of initial conditions (0 < ∅_1_(0), ∅_2_(0)≤2π), σ12ss can reach one of the solutions $\pm \frac{2n_{1}\pi }{\omega_{1}}=\pm \frac{2n_{2}\pi }{\omega_{2}}$. For the following set of parameters, simulated results show that σ12ss reaches one of the following three solutions $0,\pm \frac{2\pi }{\omega_{1}}or\pm \frac{4\pi }{\omega_{2}}or\pm 1.257$. Simulation parameters: ω_1_ = 5, ω_2_ = 10, *A* = 0.2, θ = 2.9644.

From the following Figure 6A it can be verified that ψ_12_ and σ_12_ attain $\frac{\theta }{\omega_{1}\omega_{2}}=\frac{-1.8968}{5\times 10}\approx -0.038$ and zero solution (the desired solution as mentioned before) respectively for the initialization specified in the figure caption. To check the dependency of σ12ss on the initial values of ∅_1_ and ∅_2_, ∅_1_(0) and ∅_2_(0) space is discretized at △∅_1_(0) = △∅_2_(0) = 0.1, in the interval 0 < ∅_1_(0), ∅_2_(0) ≤ 2π and eqs. 8a1, 8a2, 8b1, 8b2 is simulated for 200 s to ensure the steady-state to be achieved for various combinations of ∅_1_(0) and ∅_2_(0). From the following Figure 6B it can be observed that σ12ss achieves the following solutions: $0,\pm \frac{2\pi }{\omega_{1}}$; for *n*_1_ = 0, 1 or $n_{2}=0,2\left(=\frac{\omega_{2}n_{1}}{\omega_{1}}=2n_{1}\right)$ independent of θ.

#### Three Hopf Oscillators Coupled Through Power Coupling

We now explore the solution space numerically for a three-oscillator system. When three Hopf oscillators are coupled according to the eq. 10 or 11 (*N* = 3) with $\theta_{ij}^{\prime }$s, chosen as θ_*ij*_ = θ_*i*_ − θ_*j*_∀ 0 < θ_*i*_, θ_*j*_ ≤ 2π (i.e., θ_*ij*_ = −θ_*ij*_), at steady state σ_*ij*_ achieves one of the possible solutions depending on the initializations (∅_*i*_(0)′*s*). The $\dot{\emptyset_{i}}$ dynamics of the three oscillators can be expressed in the following form.

(14a) ∅1.=ω1+A12r2ω1ω2r1sin⁡ω1(∅2ω2-∅1ω1+θ12ω1ω2)+A13r3ω1ω3r1sin⁡ω1(∅3ω3-∅1ω1+θ13ω1ω3)

(14b) ∅2.=ω2+A23r3ω2ω3r2sin⁡ω2(∅3ω3-∅2ω2+θ23ω2ω3)+A21r1ω1ω3r2sin⁡ω2(∅1ω1-∅2ω2+θ21ω1ω2)

(14c) ∅3.=ω3+A31r1ω3ω1r3sin⁡ω3(∅1ω1-∅3ω3+θ31ω1ω3)+A32r2ω3ω2r3sin⁡ω1(∅2ω2-∅3ω3+θ32ω2ω3)

At steady-state $\dot{\psi_{ij}}=0$, where $\psi_{ij}=\frac{\emptyset_{i}}{\omega_{i}}-\frac{\emptyset_{j}}{\omega_{j}}$ or $\dot{\psi_{ij}}=\frac{\bar{\emptyset_{i}}}{\omega_{i}}-\frac{\bar{\emptyset_{j}}}{\omega_{j}}$: Assuming μ = 1, *A*_*ij*_ = *A* and *e*^*i*θ_*ij*_^ is a Hermitian matrix (*i*.*e*., *e*^*i*θ_*ij*_^ = *e*^−*i*θ_*ji*_^),

ψ12ss.=-1ω1sin⁡ω1(ψ12ss-θ12ω1ω2)+1ω1sin⁡ω1(ψ31ss-θ31ω1ω3)+1ω2sin⁡ω2(ψ23ss-θ23ω2ω3)-1ω2sin⁡ω2(ψ12ss-θ12ω1ω2)=0

ψ23ss.=-1ω2sin⁡ω2(ψ23ss-θ23ω2ω3)+1ω2sin⁡ω2(ψ12ss-θ12ω1ω2)+1ω3sin⁡ω3(ψ31ss-θ31ω1ω3)-1ω3sin⁡ω3(ψ12ss-θ12ω1ω2)=0

ψ31ss.=-1ω3sin⁡ω3(ψ31ss-θ31ω1ω3)+1ω3sin⁡ω3(ψ23ss-θ23ω2ω3)+1ω1sin⁡ω1(ψ12ss-θ12ω1ω2)-1ω1sin⁡ω1(ψ31ss-θ31ω1ω3)=0

The trivial and desirable solution set for the above equation is:

$\psi_{12}^{*}$=$\frac{\theta_{12}}{\omega_{1}\omega_{2}}$, $\psi_{23}^{*}$=$\frac{\theta_{23}}{\omega_{2}\omega_{3}}$,$\psi_{31}^{*}$=$\frac{\theta_{31}}{\omega_{1}\omega_{3}}$ or $\sigma_{12}^{*}$=σ23*=$\sigma_{31}^{*}$=0

However, like before in eq. 13a, there are several spurious solutions for which the entire right-hand sides of eqs. 14a, 14b, 14c are zero, without each of the individual terms being zero separately. So, σ_*ij*_ can achieve any of the possible solutions satisfying the above equation. It appears that finding the spurious solutions analytically are not possible.

**FIGURE 7:**
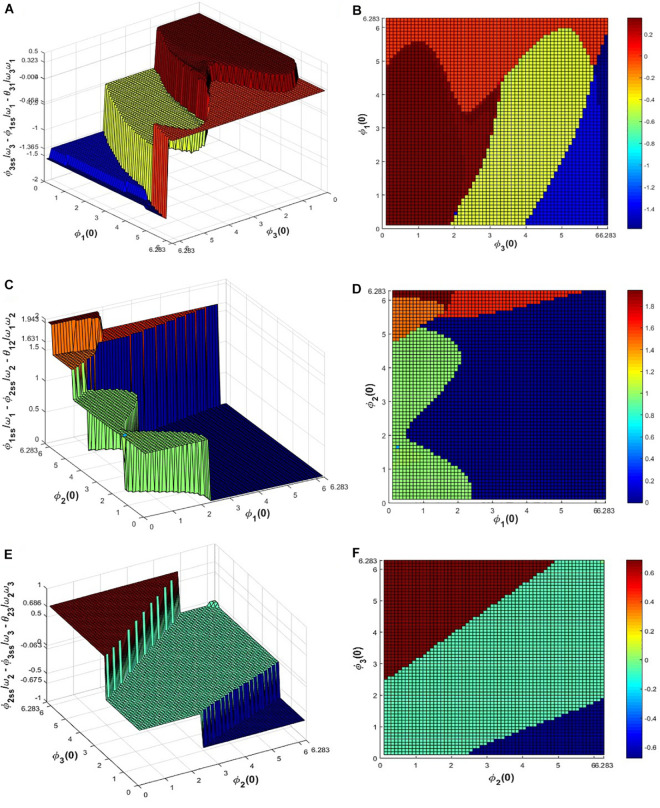
Equation 11 is simulated for 100 s for the network parameter values mentioned previously to check for what combinations of initial values of $\emptyset_{i}^{\prime }s$, σijss’s reaches, which of the solutions of eq. 14. **(A)** and **(B)** depicts that for various initial values of ∅_1_and ∅_3_ in between 0 *to*  2π, σ31ss attains 4 possible solutions: 0.3235, −0.0036, −0.4576, −1.3652, similarly from **(C-F)** it can be seen that for various initial values of ∅_1_, ∅_2_ and ∅_2_, ∅_3_ in between 0 *to*  2π, σ12ss and σ23ss attains the 0.99,  1.465, 1.6307,  1.9426, −0.0046 and 0.0633,  0.6855, −0.6751 solutions respectively, satisfying eq. 14.

To verify this numerically eq. 11 was simulated for the given set of network parameters: ω_1_ = 4, ω_2_ = 7, ω_3_ = 10, *A*_*ij*_ = *A* = 0.2, $\text{θ}=\left[\begin{array}{ccc} 0 & 1.3871 & 1.576\\ -1.3871 & 0 & 0.1889\\ -1.576 & -0.1889 & 0 \end{array}\right]$ and the initial values of $\emptyset_{i}^{\prime }s$ in the range 0 < ∅_*i*_(0) ≤ 2π with △∅_*i*_(0) = 0.1 for 100 s to let the system achieve a steady-state. It can be observed from eq. 14 that the possible solutions of σijss do not depend on θ_*ij*_ but on the natural frequencies of the oscillators (ω_*i*_). The following Figure 7 summarizes the dependency of σijss on ∅_*i*_(0) and ∅_*j*_(0) where it can be observed that σ12ss, σ23ss, and σ31ss achieve any of the 5, 3, 4 solutions respectively (mentioned in the figure caption) and particular combinations of these solutions satisfy the above equation. For a given initial value of ∅_1_, ∅_2_ and ∅_3_, σijss’s attained one of the solutions and for a confined subspace of ∅_1_(0), ∅_2_(0) and ∅_3_(0) under space 0 ≤ ∅_*i*_(0) < 2π, σijss’s attained the zero or desired solution $\left(\sigma_{ij}^{*}\right)$.

#### Hebbian Learning for the N-Oscillator System With Power Coupling

We may extend the Hebbian learning of complex coupling (eq. 5), to the case of power coupling as follows.

(15a) τWWij.=-Wij+zi(zj*)ωiωj

The polar coordinate representation:

(15b) τWAij.=-Aij+rirjωiωjcos⁡(ωi(∅iωi-∅jωj-θijωiωj))

(15c) τWθij.=ωjrirjωiωjAijsin⁡(ωi(∅iωi-∅jωj-θijωiωj))

We have limited the scope of our study only to θ_*ij*_ dynamics by assuming $\dot{A_{ij}}=0$. Let τ_*W*_ be the time constant of the above learning dynamics. It is shown both analytically and numerically that ω_*j*_ times *angle*(*W*_*ij*_) or θ_*ij*_ tries to learn ∅_*i*_ω_*j*_−∅_*j*_ω_*i*_ when the weight of the *power coupling* is trained according to eq. 15 (for proof, please refer to Supplementary Appendix 5). Similarly, as it was previously shown during the training or phase encoding of the complex coupling coefficient with very small fixed value of *A*_*ij*_ (magnitude of complex coupling coefficient), θ_*ij*_ (angle of complex coupling coefficient) learns ∅_*i*_(0)−∅_*j*_(0) (≈∅_*i*_−∅_*j*_) for arbitrary values of ω (constraining the natural frequencies to be identical). When the *power coupling* weight is updated according to eq. 15 under the same constraint as *A*_*ij*_≪1 and $\dot{A_{ij}}=0$, θ_*ij*_ learns ∅_*i*_(0)ω_*j*_−∅_*j*_(0)ω_*i*_ when there is no external input.

From eq. 11, it can be interpreted that ∅_*i*_(*t*) ≈ ω_*i*_*t* + ∅_*i*_(0) for *A*_*ij*_≪1. So, when a network of sinusoidal oscillators coupled according to eqs. 10 or 14 with the power coupling weights $\left(W_{ij}^{\prime }s\right)$ trained according to eq. 15, θ_*ij*_ learns ∅i(0)ωj-∅j(0)ωi(≈∅iωj-∅jω)i.

In such a network when individual oscillators $\left(z_{i}^{\prime }sor\emptyset_{i}^{\prime }s\right)$ are driven by complex sinusoidal forcing (Iexti=I0iei(ωit+φi)fortheithoscillator) and the power coupling weights are trained as stated, θ_*ij*_ learns φ_*i*_ω_*j*_−φ_*j*_ω_*i*_ as with *A*_*ij*_≪1, $\dot{A_{ij}}=0$ and *I*_0*i*_≫*A*_*ij*_. $\emptyset_{i}^{\prime }$s are approximately same as ω_*i*_*t* + φ_*i*_. The dynamics of such a network of supercritical Hopf oscillators is defined below:

(16) zi.=zi(μ+iωi-|zi|2)+∑j=1∋j≠iNAijeiθijωjzjωiωj+Iexti

Iexti=I0iei(ωit+φi)

Polar coordinate representation:

(17a) ri.=(μ-ri2)ri+∑j=1∋j≠iNAijrjωiωjcos⁡ωi(∅jωj-∅iωi+θijùùiωj)+I0icos⁡(ωit+φi-∅i)

(17b) ∅i.=ωi+∑j=1∋j≠iNAijrjωiωjrisinωi(∅jωj-∅iωi+θijωiωj)+I0irisin⁡(ωit+φi-∅i)

The dynamics of such a network of Kuramoto oscillators:

(18) $$\begin{array}{c} \dot{\emptyset_{i}}=\omega_{i}+\sum_{j=1∋j\neq i}^{N}A_{ij}sin\omega_{i}\left(\frac{\emptyset_{j}}{\omega_{j}}-\frac{\emptyset_{i}}{\omega_{i}}+\frac{\theta_{ij}}{\omega_{i}\omega_{j}}\right)\\ +I_{0i}sin\left(\omega_{i}t+\varphi_{i}-\emptyset_{i}\right) \end{array}$$

#### Simulated Result

**FIGURE 8:**
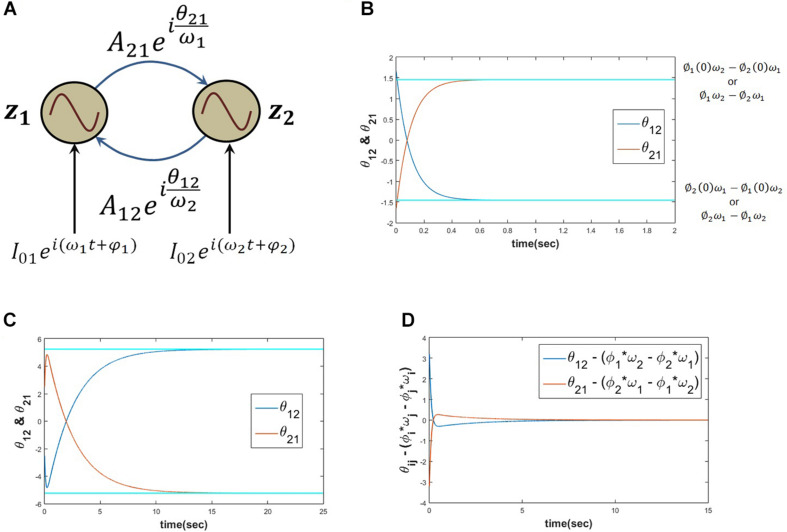
When two Hopf oscillators (*z*_1_, *z*_2_) coupled through power coupling according to eq. 16 (for *N*=2 and Iexti=0), the schematic of the network is elaborated in panel **(A)**, with the parameters ω_1_ = 5, ω_2_ = 10, *A*_12_ = *A*_21_ = 0.0001, θ_12_(0) = 1.657, θ_21_(0) = −1.657 and the power coupling weights are trained according to eq. 15, keeping the magnitude of the power coupling weight (*A*_*ij*_) constant, ω_*j*_ times the angle of power coupling, θ_*ij*_ learns ∅_*i*_(0)ω_*j*_−∅_*j*_(0)ω_*i*_ or ∅_*i*_ω_*j*_−∅_*j*_ω_*i*_. Here ∅_1_(0) = 1.2046, ∅_2_(0) = 2.7008, and τ_*W*_ = 10^3^. It is clear from the plot **(B)** that θ_12_ reaches ∅_1_ω_2_−∅_2_ω_1_ and θ_21_ reaches ∅_2_ω_1_−∅_1_ω_2_ w.r.t time. When two such Hopf oscillators as described in the previous simulation are forced by an external complex sinusoidal perturbation as described in eq. 16 (Iexti≠0) with the following parameters ω_1_ = 5, ω_2_ = 10, *A*_12_ = *A*_21_ = 0.0001, θ_12_(0) = −2.513, θ_21_(0) = 2.513, τ_*W*_ = 10^3^ and Iext1=0.5ei(5t+π4), Iext2=0.5ei(10t+π6), θ_12_, and θ_21_ learn φ_1_ω_2_−φ_2_ω_1_ = −5.7131 and φ_2_ω_1_−φ_1_ω_2_ = 5.7131 respectively **(C)**. **(D)** It can be verified that the difference between θ_12_ and ∅_1_ω_2_−∅_2_ω_1_ as well as θ_21_ and ∅_2_ω_1_−∅_1_ω_2_ becomes zero w.r.t time.

When two Hopf oscillators are coupled through power coupling with very weak coupling coefficient (*A*_*ij*_≪1) as described above in eq. 16, θ_*ij*_ learns ∅_*i*_(0)ω_*j*_−∅_*j*_(0)ω_*i*_ while there is no external input (Iexti=0) and θ_*ij*_ learns φ_*i*_ω_*j*_−φ_*j*_ω_*i*_ when *i*^*th*^ oscillator is forced by complex sinusoidal external perturbation. The following simulation results support the stated argument (Figure 8).

#### A Network of Oscillators With Adaptive Frequency and Trainable Lateral Connections

A pair of coupled adaptive Hopf oscillators driven by distinct complex sinusoidal inputs are capable of adapting their natural frequencies to the frequencies of the complex sinusoidal input signals. The trainable power coupling weight can encode the normalized phase difference between the two complex sinusoidal input signals.

When a pair of Hopf oscillators coupled through trainable power coupling coefficient (eq. 15) are driven by complex sinusoidal inputs (eq. 16), they adapt their natural frequencies according to eqs. 2b or 2c, ω_*j*_ times the angle of power coupling coefficient *W*_*ij*_ (power coupling weight from *j*^*th*^ oscillator to *i*^*th*^ oscillator) or θ_*ij*_ can learn the normalized phase difference between the complex sinusoidal input signal. If Iexti is the complex sinusoidal input signal driving *i*^*th*^ oscillator, then the normalized phase difference among them is:

ψijI=angle(Iexti)ω0i-angle(Iextj)ω0j

**FIGURE 9:**
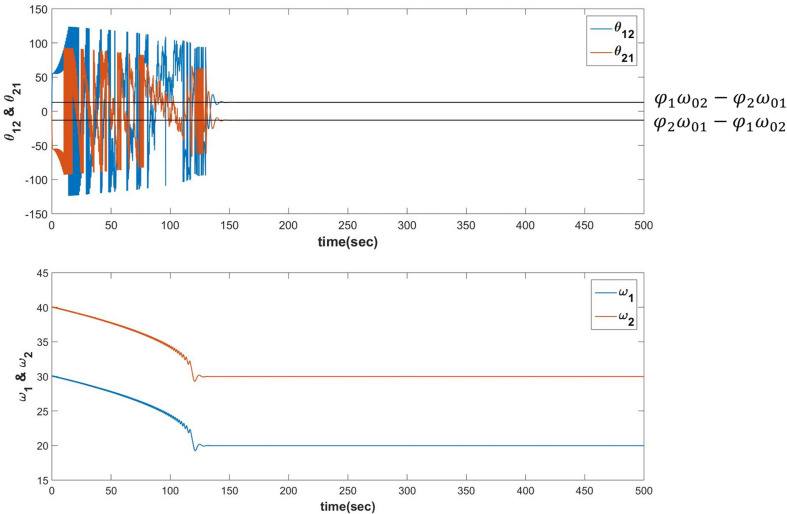
Equations 19a-c are simulated for the following set of parameters:ω_01_ = 20, ω_02_ = 30, ω_1_(0) = 30, ω_2_(0) = 40, *A*_12_ = *A*_21_ = 0.0001, θ_12_(0) = −1.7884, θ_21_(0) = 1.7884, τ_*W*_ = 10^3^, ε = 0.9 and Iext1=ei(20t+π4), Iext2=ei(30t+π6). It can be verified that θ_*ij*_ learns φ_*j*_ω_0*i*_−φ_*i*_ω_0*j*_ after the $\omega_{i}^{\prime }$s learns the corresponding $\omega_{0i}^{\prime }$s.

When the following dynamics is simulated for *N*=2 it can be verified that θ_*ij*_ learns φ_*j*_ω_0*i*_−φ_*i*_ω_0*j*_ after ω_*i*_, the natural frequency of *i*^*th*^ oscillator learns the frequency of the input signal ω_0*i*_ (described in Figure 9).

(19a) zi.=zi(μ+iωi-|zi|2)+∑j=1∋j≠iNAijeiθijωjzjωiωj+εIexti

Iexti=I0iei(ω0it+φi)

(19b) τWWij.=-Wij+zi(zj*)ωiωj

(19c) $$\dot{\omega_{i}}=-\varepsilon I_{0i}\sin\left(\emptyset_{i}-\omega_{0i}t-\varphi_{i}\right)$$

#### A Network for Reconstructing a Signal by a Fourier-Like Decomposition

In the previous sections, we have described a network of oscillators in which the natural frequencies and lateral connections can be trained. We now add a feature to the network of Section “A Network of Oscillators With Adaptive Frequency and Trainable Lateral Connections” and make it learn an unknown signal by performing a Fourier-like decomposition.

**FIGURE 10:**
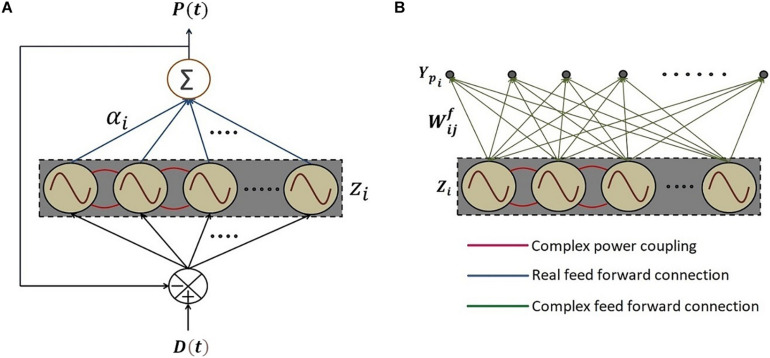
The schematic of the network where **(A)** a reservoir of N number of adaptive Hopf oscillators similar to the network proposed by Righetti et al. (2005) with additional asymmetric power coupling connections between the oscillators, each of which is driven by the same error signal *e*(*t*) between the *D*(*t*), teaching time-series signal and *P*(*t*), the linear summation of the oscillations of each of the oscillators in the reservoir through the real feed-forward connection weights, elaborated in eq. 20. **(B)** The network architecture of 2^nd^ phase of training with the same reservoir of oscillators with tuned natural frequencies and trained power coupling weights in the 1^st^ phase of training. *Y*_*p_i_*_ is the predicted output signal at the *i*^*th*^ output node. Complex feed-forward weights $W_{ij}^{f\prime }s$ connecting *j*^*th*^ oscillator to the *i*^*th*^ output node, are trained in batch mode according to eq. 23.

To this end, we construct a reservoir of Hopf oscillators (Figure 10A). It consists of a network of Hopf oscillators with trainable lateral connections (eq. 15) and trainable natural frequencies (eq. 20b). In addition, there exists a linear weight stage between the oscillators and the output layer. The natural frequencies of the oscillators adapt themselves to the nearest significant component in the input signal. The lateral connections involving power coupling, encode the (normalized) phase relationships among the oscillators. The output weights represent the amplitudes of the oscillatory components corresponding to the oscillators. A similar network architecture and dynamics for adaptable central pattern generator (aCPG) has been proposed by Righetti et al. (2005), the detailed comparison of which is discussed in Section “Discussion.”

To reconstruct the teaching time-series signal at the output summation node, the error signal that drives each of the oscillators drives the phase offset to the desired phase offset associated with the corresponding frequency present in the teaching time-series signal. The activation and learning dynamics of the network are given below.

(20a) zi.=zi(μ+iωi-β1|zi|2)+∑j=1∋j≠iNWijzjωiωj+εe(t)

$$W_{ij}=A_{ij}e^{i\frac{\theta_{ij}}{\omega_{j}}}$$

(20b) $$\dot{\omega_{i}}=-\eta_{\omega }e\left(t\right)\sin\emptyset_{i}$$

(20c) $$\dot{\alpha_{i}}=\eta_{\alpha }e\left(t\right)r_{i}\cos\emptyset_{i}$$

(20d) τWWij.=-Wij+zi(zj*)ωiωj ∋Aij.=0

(20e) e(t)=D(t)-P(t)

$$P\left(t\right)=\sum_{i=1}^{N}\alpha_{i}\cos\emptyset_{i}$$

**FIGURE 11:**
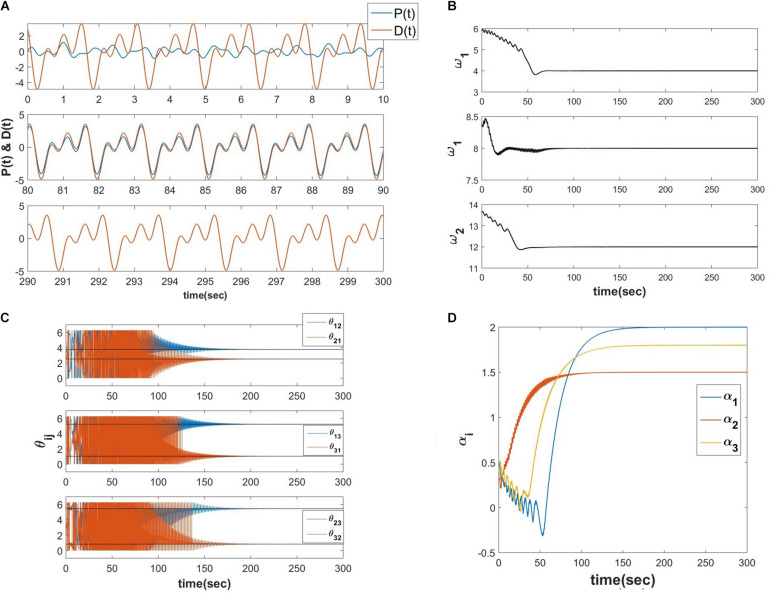
Equations 20a-e is simulated with the following parameters $D\left(t\right)=2\cos\left(4t+\frac{\pi }{2}\right)+1.5\cos\left(8t+\frac{\pi }{5}\right)+1.8\cos\left(12t+\frac{\pi }{12}\right)$, *A*_*ij*_ = 10^−5^, η_ω_ = 0.1, η_α_ = 10^−4^, τ_*W*_ = 10^4^, ε = 0.5, *N* = 3, *dt* = 10^−3^ *sec*. **(A)** The network learned signal at the output summation node *P*(*t*) and the *D*(*t*) signal at various 10 s of time interval. **(B)** The natural frequencies of the three oscillators learn the three frequency components present in the teaching signal. **(C)** ω_*j*_ times the angles of the complex power coupling weights (θ_*ij*_) learn φ_*i*_ω_*j*_−φ_*j*_ω_*i*_. **(D)** The real feed-forward weights α_*i*_’s learn *a*_*i*_’s.

Let *D*(*t*) be the teaching time-series signal with a finite number of frequencies ($D\left(t\right)=\sum_{i=1}^{N}a_{i}\cos\left(\omega_{i}t+\varphi_{i}\right)$ where φ_*i*_ be the phase offset associated with *i*^*th*^ frequency component), *z*_*i*_ = *r*_*i*_*e*^*i*∅_*i*_^, $\alpha_{i}^{\prime }s$ are the real feed-forward weights from *i*^*th*^ oscillator to the output summation node, τ_*W*_, η_ω_ and η_α_ are the time constant of the learning dynamics for power coupling coefficient, the learning rate of the natural frequency of the oscillators and the real feed-forward weights from oscillators to the output summation node respectively, *P*(*t*) is the reconstructed signal at the output summation node. The numerical simulation of the proposed network (Figure 10A describes the schematic) is elaborated in the following Figure 11.

We propose a similar network for Fourier decomposition of complex time series signal with a finite number of frequencies (assuming $D\left(t\right)=\sum_{i=1}^{N}a_{i}e^{i\left(\omega_{i}t+\varphi_{i}\right)}$), which is an extension of the network proposed in section “A Network of Oscillators With Adaptive Frequency and Trainable Lateral Connections.” The proposed network is comprised of a reservoir of complex adaptive Hopf oscillators coupled through trainable power coupling weights, and driven by distinct complex sinusoidal inputs with arbitrarily different frequencies. The network is capable of learning the frequencies and encode the normalized phase relationship among the oscillatory components of the input signal. It is driven by the error signal between the complex teaching signal and the linear summation of complex activations of the Hopf oscillators. The dynamics of the network is described in the following equations.

(21a) zi.=zi(μ+iωi-β1|zi|2)+∑j=1∋j≠iNWijzjωiωj+εe(t)

$$W_{ij}=A_{ij}e^{i\frac{\theta_{ij}}{\omega_{j}}}$$

(21b) $$\dot{\omega_{i}}=-\eta_{\omega }\left(real\left(e\left(t\right)\right)\sin\emptyset_{i}-img\left(e\left(t\right)\right)\cos\emptyset_{i}\right)$$

(21c) $$\dot{\alpha_{i}}=\eta_{\alpha }\left(real\left(e\left(t\right)\right)r_{i}\cos\emptyset_{i}+img\left(e\left(t\right)\right)r_{i}\sin\emptyset_{i}\right)$$

(21d) τWWij.=-Wij+zi(zj*)ωiωj∋Aij.=0

(21e) e(t)=D(t)-P(t)

$$P\left(t\right)=\sum_{i=1}^{N}\alpha_{i}z_{i}$$

**FIGURE 12:**
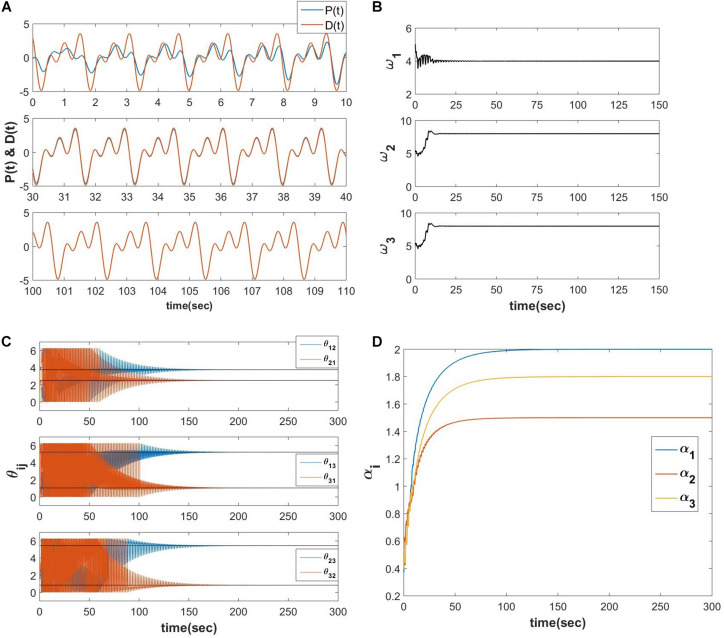
Equations 21a-e are simulated for a teaching signal constituting three frequency components with the parameters $D\left(t\right)=2e^{i\left(4t+\frac{\pi }{2}\right)}+1.5e^{i\left(8t+\frac{\pi }{5}\right)}+1.8e^{i\left(12t+\frac{\pi }{12}\right)},A_{ij}=10^{-5},\eta_{\omega }=0.1,\eta_{\alpha }=10^{-4},\tau_{W}=10^{4},\varepsilon =0.5,N=3,dt=10^{-3}sec$. **(A)** The real part of the network learned signal at the output summation node *P*(*t*) and the *D*(*t*) signal at various 10 s of time interval. **(B)** The natural frequencies of the three oscillators learn the three frequency components present in the teaching signal. **(C)** ω_*j*_ times the angles of the complex power coupling weights (θ_*ij*_) learn φ_*i*_ω_*j*_−φ_*j*_ω_*i*_. **(D)** The real feed-forward weights α_*i*_’s learn *a*_*i*_’s.

The schematic of the network is identical to the network described in Figure 10A, and the simulated result for a *D*(*t*), which contains three frequency components is as follows (Figure 12).

#### A Generative Network Which Is Capable of Modeling EEG Signals

The proposed network is capable of modeling an arbitrary number of signals with an overlapping frequency spectrum. It is trained in two phases. In the first phase, a network exactly similar to the one in the previous section (Section “A Network for Reconstructing a Signal by a Fourier-Like Decomposition”) is used to encode the constituting frequency components of one of the input signals. During this phase, the natural frequencies ($\omega_{i}^{\prime }$s), the real feed-forward weights ($\alpha_{i}^{\prime }s$) as well as the power coupling weights $\left(W_{ij}^{\prime }s\right)$ of a network of *N* Hopf oscillators are trained using the same learning rule described in the previous section (eq. 20). One key difference compared to the previous section is that the teaching signal in the present scenario is aperiodic (frequency spectrum is continuous) and has a finite duration. To overcome this issue, the limited duration teaching EEG signal is presented repeatedly to the network over multiple epochs. This helps the network to learn some sort of Fourier decomposition of the teaching signal.

In the second phase of training, the trained reservoir of oscillators tries to reconstruct *M* number of signals at the corresponding *M* output nodes assuming these *M* signals are the outcome of the same underlying process (producing signals with frequencies confined to a certain frequency band) by training the complex feed-forward weights connecting *N* oscillators of the reservoir to the *M* output nodes. The schematic of the network architecture (Figure 10B) and the corresponding learning rules are described below.

#### First Phase of Learning

During the first phase of learning, an identical network with identical learning dynamics as described by eq. 20 is used. The only fundamental difference between the previous and present scenario is that *D*(*t*) is a limited duration aperiodic or quasiperiodic signal compared to the previous case where *D*(*t*) was an infinite duration periodic or quasiperiodic signal. i.e., there can be an infinite number of frequency components present in *D*(*t*) as the frequency spectrum of an aperiodic signal is continuous (refer to Figure 14A). So, the Fourier decomposition using the proposed network can be accomplished by discretely sampling the continuous frequency spectrum.

#### Second Phase of Learning

In this phase, the supervised batch mode of learning is used to train the complex feed-forward weights, *W^f^*, defined as follows. Here *K*_*ij*_ and ζ_*ij*_ are respectively the magnitude and angle of the complex feed-forward weights. The derivation for the batch update rule for *K*_*ij*_ and ζ_*ij*_ as described in eq. 22 is given in the Supplementary Appendix 6.

(22a) $$W_{ij}^{f}=K_{ij}e^{i\zeta_{ij}}$$

(22b) Ypi(t)=real(∑j=1NWijfei∅j)

(22c) △Kij=(-1)ηK∑t(Ydi(t)-Ypi(t))cos⁡(∅j(t)+ζij)

(22d) △ζij=(-1)ηζ∑t(Ydi(t)-Ypi(t))(-Kijsin⁡(∅j(t)+ζij))

#### Simulation Results

**FIGURE 13:**
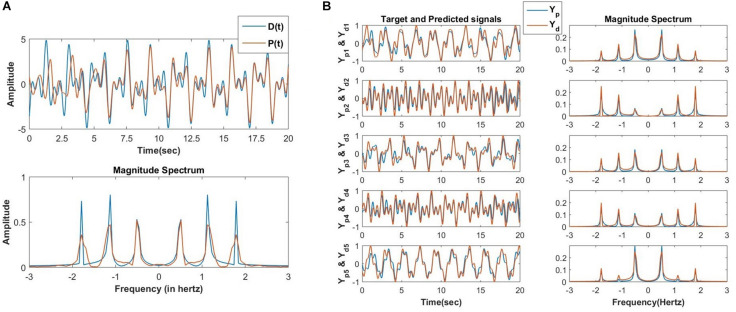
**(A)** In the first phase of training the oscillatory reservoir network (Figure 10A) with three oscillators is trained using the following teaching signal and learning parameters: *D*(*t*) = *A*_1_*cos*⁡(ω_1_*t* + ∅_1_) + *A*_2_*cos*⁡(ω_2_*t* + ∅_2_) + *A*_3_*cos*⁡(ω_2_*t* + ∅_2_), *A*_*ij*_ = 10^−1^, η_*w*_ = 10^−1^, η_α_ = 10^−4^, τ_*w*_ = 10^4^, ϵ = 0.5, *N=3*, *dt* = 10^−3^*sec*, *n*_*epoch*_ = 15. The network produced and the original teaching signal and their magnitude spectrum respectively after the last epoch (15) of training. **(B)** After the second phase of training, the network (Figure 10B) is able to reconstruct the *M=5* output signals (*Y_d_i__ for i* = 1 *to M*) with identical frequency components but randomly chosen amplitude (*A*_*i*_) and phase offset (∅_*i*_) with high accuracy. Learning parameters: η_*K*_ = 3 × 10^−5^, η_*ζ*_ = 10^−6^, no. of epochs for the batch mode of learning = 10000.

At first, the proposed network is simulated to model an arbitrary number of quasiperiodic signals with identical frequency components. The desired signal (*D*(*t*)) consisting of three frequency components (provided $\frac{\omega_{i}}{\omega_{j}}$ is not an integer) with a duration of 20 s is used for learning. The network output *P*(*t*) signal (after the first phase of learning) and the respective frequency spectrum is plotted in Figure 13A. After the second phase of training, the desired and the network reconstructed signals are plotted in Figure 13B, and the training parameters are described. One crucial condition has to be met in order to achieve an accurate reconstruction: All the oscillators in the reservoir has to be initialized at *z*_*i*_(0) = 1.

**FIGURE 14:**
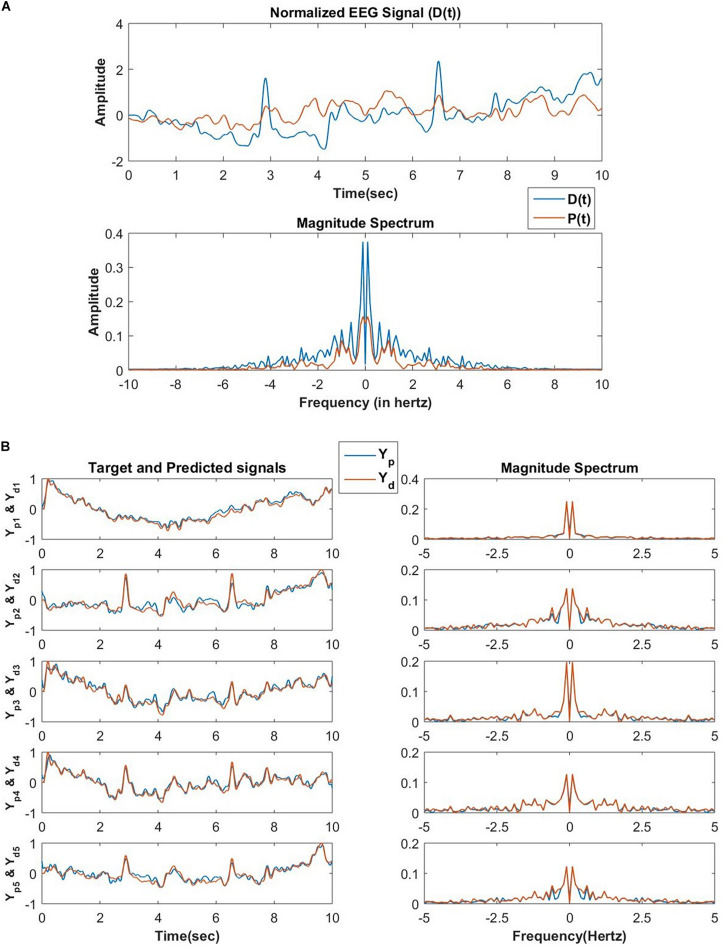
**(A)** The reconstructed signal by the network of 100 oscillators after 30 epochs, the teaching signal, and the respective frequency spectrum in the first phase of training. In this phase of training, the *D*(*t*) signal is an EEG signal of duration 10 s, and the exactly similar EEG signal is presented for consecutive 30 epochs. The learning parameters are: *A*_*ij*_ = 10^−1^ for |*j*−*i*| < 5 and *A*_*ij*_ = 0 for |*j*−*i*|≥5η_ω_ = 0.1, η_α_ = 10^−4^, τ_*W*_ = 10^4^, ε = 0.5, *N* = 100, *dt* = 10^−3^ sec *n*_*epoch*_ = 30. **(B)** After the second phase of training, the network reconstructed and the original EEG signals collected from 5 channels during the same experiment from which *D*(*t*) for the first phase of training was collected and the corresponding frequency spectrum. Learning parameters: η_*K*_ = 3×10^−5^, η_ζ_ = 10^−6^, no. of epochs for the batch mode of learning = 1000.

The network performance is tested on low pass filtered (cut off frequency of 5 Hertz) EEG data collected from a human subject while performing mind wandering task (Grandchamp et al., 2014). In the first phase, the network encodes *N* number of frequency components of an EEG signal collected from one channel of EEG recording during the mentioned experiment. There is a constraint imposed on the magnitude of the lateral power coupling weights as shown in Figure 14. This constraint allows only oscillators with nearby frequency components to interact with each other as initially, the natural frequencies of these oscillators are sorted at an increasing order after sampling from a uniform probability distribution ranging between 0 and 5 Hertz.

In the second phase, the network tries to reconstruct the EEG signals collected simultaneously from the other channels during the same experiment. A set of EEG signals from 5 other channels are reconstructed by the network. The reconstruction accuracy of the modeled EEG signals depends on the number of oscillators in the oscillatory layer and the number of desired signals to be reconstructed. Using complex weights for the feed-forward connections is a key factor as it helps to learn the amplitude, as well as phase of the Fourier decomposition of the EEG signal to be reconstructed, provided the frequency components are already learnt in the encoding phase.

## Discussion

The unique contribution of the proposed network of complex neural oscillators is the notion of power coupling by which it becomes possible to achieve a stable normalized phase relationship between two oscillators with arbitrary natural frequencies. With such a feature as the backbone, it is possible to construct a network of oscillators that can learn to reconstruct multiple time series signals. Another positive feature of the proposed model is the biological feasibility of the learning mechanisms. The lateral connections in the oscillatory reservoir are trained by a Hebb-like rule, while the forward connections are trained by a modified delta rule adapted to the complex domain. Experimental observations reveal that local interaction occurs at the single neuron level through single synapse whereas long-range interaction occurs between various cortical areas mediated by multiple synapses (Supplementary Figure 6). At single-cell level the synaptic property can define the relative phase difference between tonically firing presynaptic and postsynaptic neurons. Neuronal population-level interaction between various cortical areas can facilitate cross-frequency coupling ensuring a certain phase relationship (Hyafil et al., 2015) between the LFP activity of different frequencies of the respective cortical areas.

### Other Complex Valued Network Models

There is a vast literature pertaining to complex-valued neural network models, which are produced by extending their real-valued counterparts to the complex domain. Complex feed-forward networks trained by the complex backpropagation algorithm, complex Hopfield network, complex self-organizing maps are examples of such models. Complex formalism plays a key role in linear systems theory in which both systems and signals can be represented as complex functions. As a special case of this, in electrical circuit theory, oscillations are modeled as complex numbers called phasors, that represent the phase of the oscillations. Therefore, there is a longstanding association between complex numbers and oscillations. Nevertheless, complex-valued neural network literature did not seem to have exploited this association. For the most part, complex-valued neural networks operate like 2n dimensional versions of their n-dimensional real-valued counterparts. The proposed model attempts to take an important step in filling this lacuna. By describing oscillations in the complex plane, and invoking the power coupling principle, it is able to elegantly overcome some of the difficulties involved in harnessing the dynamics of multi-oscillator networks with real coupling.

Network models of complex-valued oscillators have been described before. For example, subsequent studies by Hoppensteadt and Izhikevich (1996, 2000) have shown that weakly connected network of complex oscillators with identical natural frequencies and self-adjoint matrix of complex coupling coefficient can achieve any arbitrary phase difference between two oscillators. It has also been shown that such network can store and retrieve at least one memory pattern in terms of the phase difference of the oscillators by enabling the Hebbian-like learning rule of the complex coupling coefficient as given in eq. 5. Recent study by Kim and Large (2021) has extended the steady-state stability analysis of such a pair of oscillators to the special case of frequency detuning (ω_1_ ≠ ω_2_) for a range of parameter space. In the same study (Kim and Large, 2021), the authors have proposed a generalized framework for a pair of coupled multifrequency complex oscillators (Hoppensteadt and Izhikevich, 1997) with a Hebbian like learning rule for the complex coupling coefficient. Unlike the proposed power coupling strategy, the coupling is defined for oscillators with natural frequencies near the resonant condition: *m*ω_1_ = *n*ω_2_, where *m* and *n* are integer numbers. Similar steady-state stability analysis of such a pair of complex oscillators (for *n=2*, *m=1* and higher order) shows that the angle of the complex coupling coefficient (θ_*ij*_) learns *m*∅_1_−*n*∅_2_ for Ω_12_ = *m*ω_1_−*n*ω_2_ = 0, and increases/decreases linearly or rotates for Ω_12_ ≠ 0 at steady state. However, neither of the study extended the result to the case of interacting oscillators with very different natural frequencies (|*m*ω_1_−*n*ω_2_| > *ϵ*, where ϵ is a small positive number), as we have done using power coupling. Table 1 summarizes the comparison between four different coupling strategies between a pair of complex Hopf oscillators. Comparison is made principally w.r.t two dynamical properties: entrainment and synchronization and Hebbian like plasticity of the coupling coefficient and the coupling term influencing the oscillator dynamics. Frequency entrainment is the dynamical phenomena when the natural frequency of two coupled autonomous oscillators converges to an intermediate frequency (Pikovsky et al., 2001). Whereas the synchronization has been defined here in a more general sense: any coupled oscillators can exhibit synchronization if they maintain any of the phase relationships (∅_*i*_−∅_*j*_, *m*∅_*i*_−*n*∅_*j*_, $\frac{\emptyset_{i}}{\omega_{i}}-\frac{\emptyset_{j}}{\omega_{j}}$) constant at steady-state with or without being entrained. A network of complex-valued oscillators that can store patterns as oscillatory states was described in Chakravarthy and Ghosh (1996). The model also proposed a complex form of Hebb’s rule, similar to the one used in the current study. However, the model of Chakravarthy and Ghosh (1996) was limited in its capability by the fact that all the oscillators in the model have a common frequency. A representation of synaptic strength using a complex weight, instead of the usual real weight, has an added advantage in representing the temporal relationships underlying neural dynamics. We have recently shown (Chakravarthy, 2020) that the imaginary part of the complex weight captures the temporal asymmetry between the activities of pre- and post-synaptic neurons in a manner akin to the weight kernel of Spike Time Dependent Plasticity (STDP) mechanism (Bi and Poo, 1998).

**TABLE 1:**
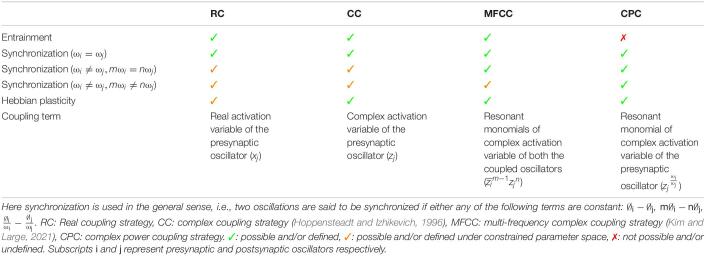
The brief comparison among the various coupling strategies between a pair of complex Hopf oscillators.

It may be said that the network of Section “A Generative Network Which Is Capable of Modeling EEG Signals” achieves a discrete approximation of the continuous spectra of the signals that are reconstructed by the network. The greater the number of oscillators in the network, the tighter is the approximation. In this approximation, the trainable real feed-forward weights (α_*i*_) of the network learn the amplitude spectrum of *D*(*t*). The angle of the power coupling weight learns normalized phase differences, $\frac{\varphi_{i}}{\omega_{i}}-\frac{\varphi_{j}}{\omega {}_{j}}$.

### Analogous Trainable Central Pattern Generator Model and Comparison

It has previously been shown in Righetti et al. (2005) that, when a network of supercritical Hopf oscillators driven by the reconstruction error signal, individual oscillators are tuned to the nearest frequency components in the target time series. In the network (net-1: schematic is described in Figure 2, and the network dynamics and the learning dynamics is given in eqs. 4 to 8 of Righetti et al., 2005) one shortcoming is that there is no way to recreate the teaching time-series signal (*P*_*teach*_(*t*)) back without the error feedback loop as with the learnt αis′ and ωis′, when the network is reset (i.e., ris′ and ∅is′ are reset) there is no external forcing to drive the phase offset of each oscillator to the desired phase offsets $\left(\varphi_{i}^{\prime }\text{s}\right)$. To overcome this issue, the authors have proposed a phase signal of frequency same as *i*^*th*^ oscillator but same phase as the oscillator with the lowest frequency as a signal communicated by the oscillator with the lowest frequency to the *i*^*th*^ oscillator, which makes those two oscillators maintain a certain normalized phase difference $\left(\frac{\emptyset_{i}}{\omega_{i}}-\frac{\emptyset_{0}}{\omega_{0}}\right)$. However, to learn/store the phase relationship between the lowest frequency component and the *i*^*th*^ frequency component in the teaching signal a “phase variable” considered as a third variable of the oscillator dynamics has been defined which learns the difference between phase signal from the oscillator with the lowest frequency and its own phase. The main issue with this network is that the network can only learn Fourier decomposition (frequency, magnitude and relative phase offset among the constituting frequency components) of a periodic signal which has frequency components of the form ω_*i*_ = *n*ω_0_, ω_0_ being the fundamental frequency of the periodic signal as *n* ∈ *N* as the phase signal *R_i_* from the 0^*th*^ oscillator is interacting with the *x* dynamics of the 0^*th*^ oscillator through real coupling constant. Furthermore, there is an ambiguity in defining the lowest frequency component or fundamental frequency of the periodic teaching signal as the dynamics do not itself identify the oscillator, which learns the lowest frequency component in the teaching signal.

The above arrangement of representing phase difference using an explicit phase parameter located in the *i*^*th*^ oscillator has another drawback of Righetti et al. (2005). Phase difference between two oscillators is a property that arises out of their interactions. Ideally, it must be represented as a property of the interaction strength, which in our case is the complex weight parameter. In Righetti et al. (2005), since phase difference information *between* two oscillators has to be encoded *within* one of the oscillators, in a rather awkward fashion, each oscillator is restricted to encode the phase difference only with one “standard” oscillator – the oscillator corresponding to the fundamental frequency. On the contrary, in our proposed model, (normalized) phase difference arises jointly out of the power coupling and the corresponding complex weight, there is no need for the above-mentioned restrictions. The oscillators can independently control their (normalized) phase differences with other oscillators via their mutual coupling parameters.

To overcome these issues, the proposed network as described in section “A Generative Network Which Is Capable of Modeling EEG Signals” is a similar network where the reservoir of complex supercritical Hopf oscillators is coupled through power coupling to preserve the phase relationship among the constituting frequency components in the teaching signal. When second network architecture proposed by Righetti et al. (schematic of which is described in Figure 3 and the dynamics is given in eqs. 13-20 of Righetti et al. (2005) tries to learn Fourier decomposition of a teaching signal the natural frequencies of the oscillators and the real feed-forward weights learns the magnitude spectrum of the teaching signal. During learning the error signal, *F*(*t*) drives the phase offset (∅_*i*_−ω_*i*_*t*) of each of the oscillators to the desired phase offset (φ_*i*_) can be retrieved from the phase spectrum of the teaching signal. Thus, the same network deliberated by eq. 20 with the oscillators coupled through power coupling with fixed *A*_*ij*_ (≪μ) ensures θ_*ij*_ to learn φ_*i*_ω_*j*_−φ_*j*_ω_*i*_.

One issue while retrieving the stored oscillatory pattern is the dependence on the initial state of the oscillators to achieve the desired solution (see section “Hebbian Learning for the N-Oscillator System With Power Coupling”). We observed that when ∅_i_(0)′s are sampled from space ∧, subspace of ∧ = {*x* ∈ *R^N^*|0 ≤ *x* ≤ 2*π*}, the network attains the desired solution at a steady-state. However, there are other plausible solutions or spurious states the network can acquire. The desired solution of σij*(=0) and the spurious solutions are independent of the network parameters like *A*_*ij*_ (for *A*_*ij*_ = *A*) and θ_*ij*_ but has a dependence on the natural frequency of the oscillators. However, the solutions of ψ_*ij*_ are dependent on both network parameters (θ_*ij*_ and ω_*i*_, assuming *A*_*ij*_ = *A*). The dependency of the boundary of space ? or the boundary of the other subspaces (initializing ∅_i_ by sampling which leads the system to spurious states) on the natural frequency of the oscillators is yet to be understood. A brief comparison between aCPG model of Righetti et al. (2005) and the proposed aCPG model is summarized in the following Table 2.

**TABLE 2:**
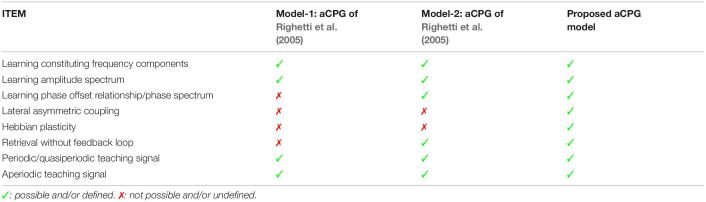
This table compares various features related to network architecture, scope of trainability, retrievability, type of teaching signals the model can learn; of the two adaptive central pattern generator (aCPG) models proposed by Righetti et al. (2005) with our proposed aCPG model.

### Large-Scale Network Models

In the present study, we use the oscillatory neural network model to reconstruct six EEG time series simultaneously. However, the proposed learning mechanisms and network architecture can be easily scaled up. It is possible to model a much larger number of EEG channels (say, 128 or 512) simply by increasing the size of the oscillatory reservoir and the number of output neurons. In the current study, the connections among the oscillators are constrained to ensure that only oscillators with nearby natural frequencies are connected. In a large-scale model, it is conceivable to associate the individual neural oscillators with anatomical locations in the brain, and constrain their coupling based on structural connectivity information from structural imaging tools like Diffusion Tensor Imaging (DTI) (Damoiseaux and Greicius, 2009). Through such developments, the present model can evolve into a class large-scale models of brain dynamics similar to The Virtual Brain (TVB) model, with some relative advantages.

The TVB model principally uses neuronal mass models like the two-variable nonlinear oscillatory models including Fitzhugh-Nagumo model, Winson-Cowan model, Wong-Wang model, Brunel-Wang model, Jensen-Rit model, Stefanescu-Jirsa model (Wilson and Cowan, 1972; Jansen and Rit, 1995; Brunel and Wang, 2003; Wong and Wang, 2006; Stefanescu and Jirsa, 2008), which can exhibit excitable dynamics and limit cycle oscillations. In these models, the oscillators are coupled by only one variable, which is analogous to “real-valued” coupling. They are trained by global optimization algorithms that adjust the coupling strengths so that the output of the oscillatory network matches the recorded brain dynamics. Furthermore, most of them do not have an explicit natural frequency as an independent parameter which is trainable depending on the external input signal. Powanwe and Longtin (2019) developed a large-scale cortical model using a network of stochastic Wilson-Cowan model with ‘smooth nonlinearity’ to model gamma-band LFP activity. The LFP activity of excitatory and inhibitory population of neurons is modeled using sinusoidal variations (*V*_*x*_(*t*) = *Z*_*x*_(*t*)cos⁡(ω_0_*t* + ∅_*x*_(*t*))). The three characteristic features, envelop (*Z*_*x*_(*t*)), constant mean frequency (ω_0_) and phase (∅_*x*_(*t*)) respectively are dependent on network parameters. ω_0_ is dependent on excitatory synaptic strength, tunable through Hebbian plasticity. Other large-scale modeling studies on the same lines (Mejias et al., 2016; Joglekar et al., 2018) focus on investigating the principal network parameters behind signal propagation or intercommunication between various cortical areas located at the various levels of hierarchy. Although these models can capture the large-scale network entrainment by the external input signal through Hebb-like plasticity of the excitatory synaptic connection, they fail to provide simplified network dynamics essential to learn three constitutive features (frequency, magnitude, and phase offset) of any input signal.

### Attractor Dynamics

The proposed network in section “Power Coupling Among N Hopf Oscillators” can reproduce a given time series signal as a linear summation of the oscillations with the same frequency components as the natural frequency of the oscillators in the reservoir and the relative phase relationships encoded in terms of the angle of the power coupling. Given the similarities between conventional attractor neural networks such as the Hopfield network, storing multiple “patterns” can be regarded as multiple time series signals with identical frequency components but non-identical relative phase relationships among them. As the relative phases among the oscillations is encoded in terms of the angle of the power coupling coefficient, the *p*^*th*^ pattern can be regarded as:

$$x_{p}=e^{i\theta_{p}}$$

where, θ_**p**_ is a N-dimensional vector, N being the no of oscillators. The power coupling coefficients are assigned as:

$$W_{ij}=ae^{i\frac{\Theta_{ij}}{\omega_{j}}}$$

Where,

eiΘ=∑pxpxp*

xp* is the complex conjugate of the transpose of *x_p_*. However, the network retains the phase relationship among the oscillators defined by the combined phase, **Θ**, instead of the individual phase relationships defined by various patterns. The model equations in the current form do not seem to support storage of multiple patterns within the oscillator layer. However, with slightly modified equations in the complex domain, it is possible to achieve multiple pattern storage (Chakravarthy and Ghosh, 1996; Chakravarthy, 2009). The proposed network architectures seem to be robust to the noise inherent to the system dynamics as well as external noise, corrupting the input signal. A brief study on the performance of key network architectures w.r.t the noise power is given in the Supplementary Figures 1-5.

### Future Perspective

In the future, we plan to extend the proposed model to create a whole class of deep oscillatory networks. The network will have an input stage that serves as an encoder that performs a Fourier-like decomposition of the input time-series signals. The network model of Section “A Network for Reconstructing a Signal by a Fourier-Like Decomposition” will play the role of this encoder. The hidden layers will consist of oscillators operating at a range of frequencies. The output layer will be a decoder that converts the oscillatory outputs of the last hidden layer into the output time series. Another interesting proposed study is to develop the network model of Section “A Network for Reconstructing a Signal by a Fourier-Like Decomposition” into a model of the tonotopic map. Electrophysiological recordings from the bat’s auditory cortex revealed a map of frequencies (a tonotopic map). Kohonen had proposed a model of the tonotopic map using a self-organizing map (Kohonen, 1998). However, this model represents frequency as an explicitly available parameter, without actually modeling the responses of the neurons to oscillatory inputs (tones). We propose that by organizing the oscillators in the reservoir model of Section “A Network for Reconstructing a Signal by a Fourier-Like Decomposition” in a 2D array with neighborhood connections, it is possible to produce a biologically more feasible tonotopic map model.

## Data Availability Statement

The original contributions presented in the study are included in the article/Supplementary Material, further inquiries can be directed to the corresponding author/s.

## Author Contributions

The simulations were done mostly by DB with the support of SP. DB wrote the main text. VC contributed to providing the key ideas, conducting initial simulations to test the hypothesis, editing the manuscript drafts, and providing insight into structure. All authors contributed to the article and approved the submitted version.

## Conflict of Interest

The authors declare that the research was conducted in the absence of any commercial or financial relationships that could be construed as a potential conflict of interest.
