# Supplementary figures and images for: A Complex-Valued Oscillatory Neural Network for Storage and Retrieval of Multidimensional Aperiodic Signals
Source: Front Comput Neurosci. 2021 May 24;15:551111. doi: 10.3389/fncom.2021.551111 (PMC8181409; doi:10.3389/fncom.2021.551111)

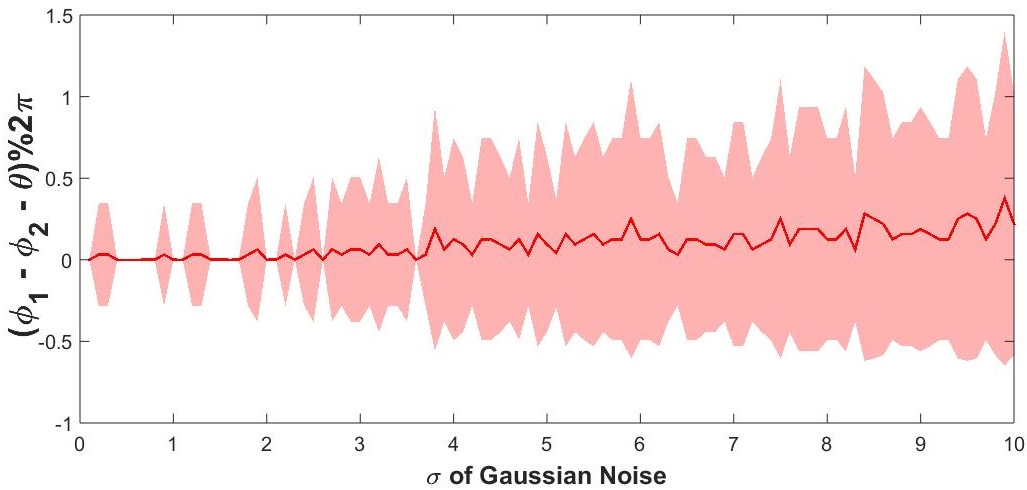

Supplement: Supplementary file 1 [file Image_1.JPEG]

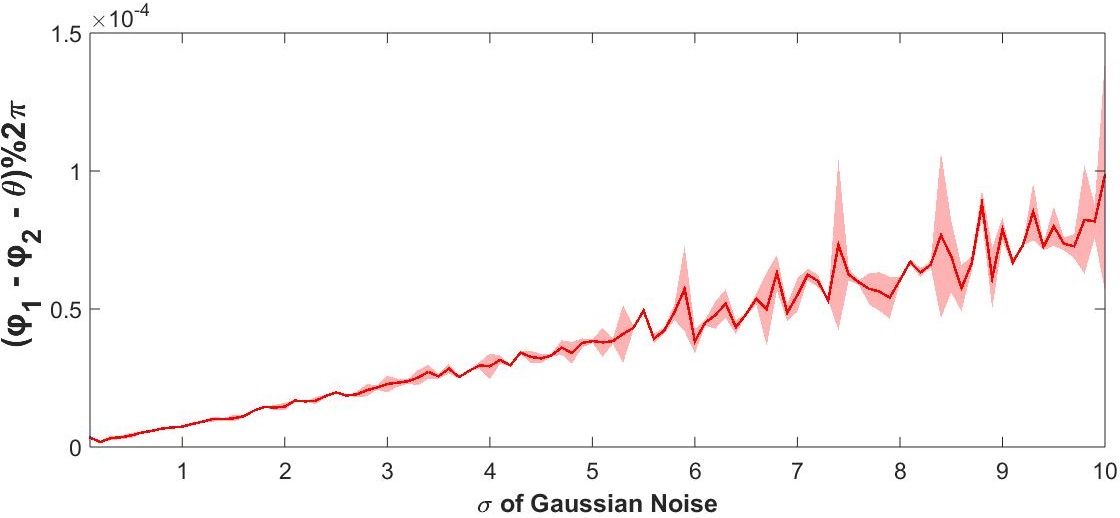

Supplement: Supplementary file 2 [file Image_2.JPEG]

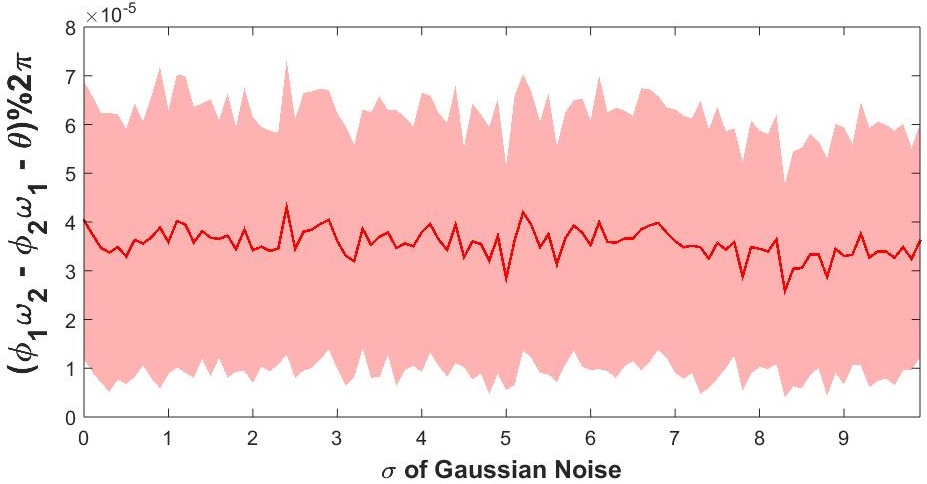

Supplement: Supplementary file 3 [file Image_3.JPEG]

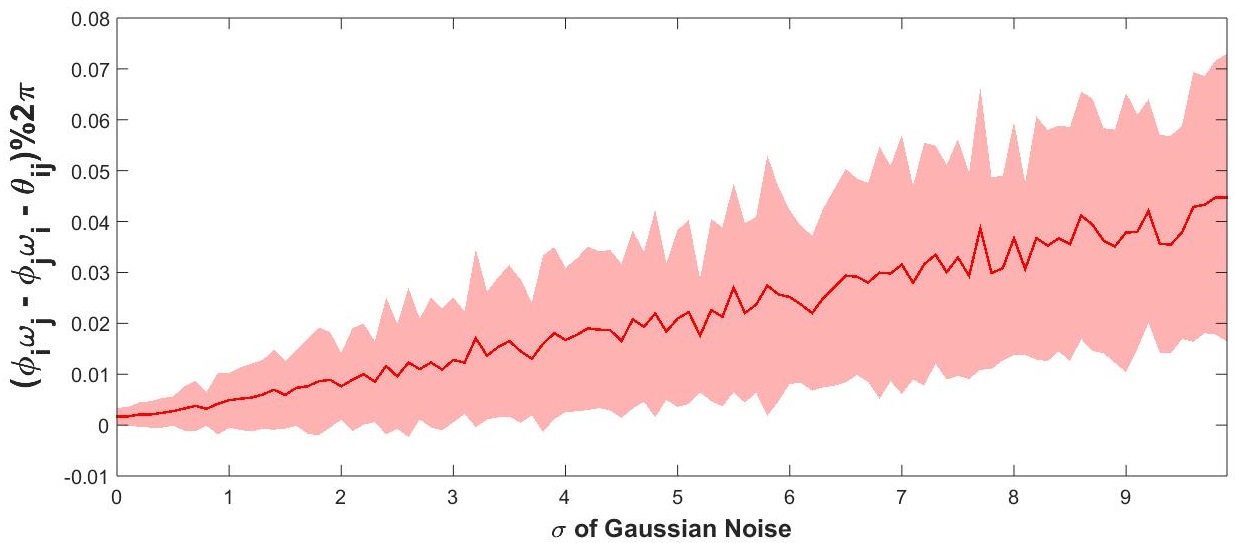

Supplement: Supplementary file 4 [file Image_4.JPEG]

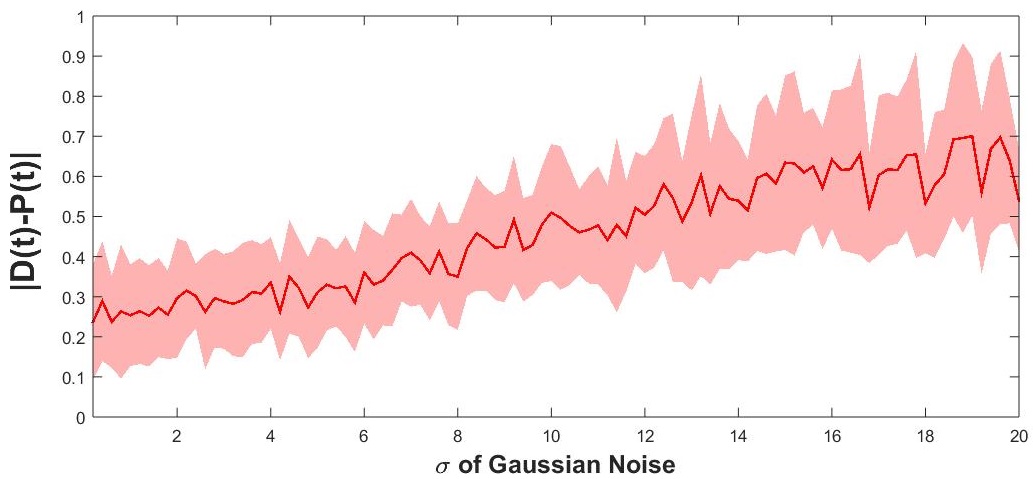

Supplement: Supplementary file 5 [file Image_5.JPEG]

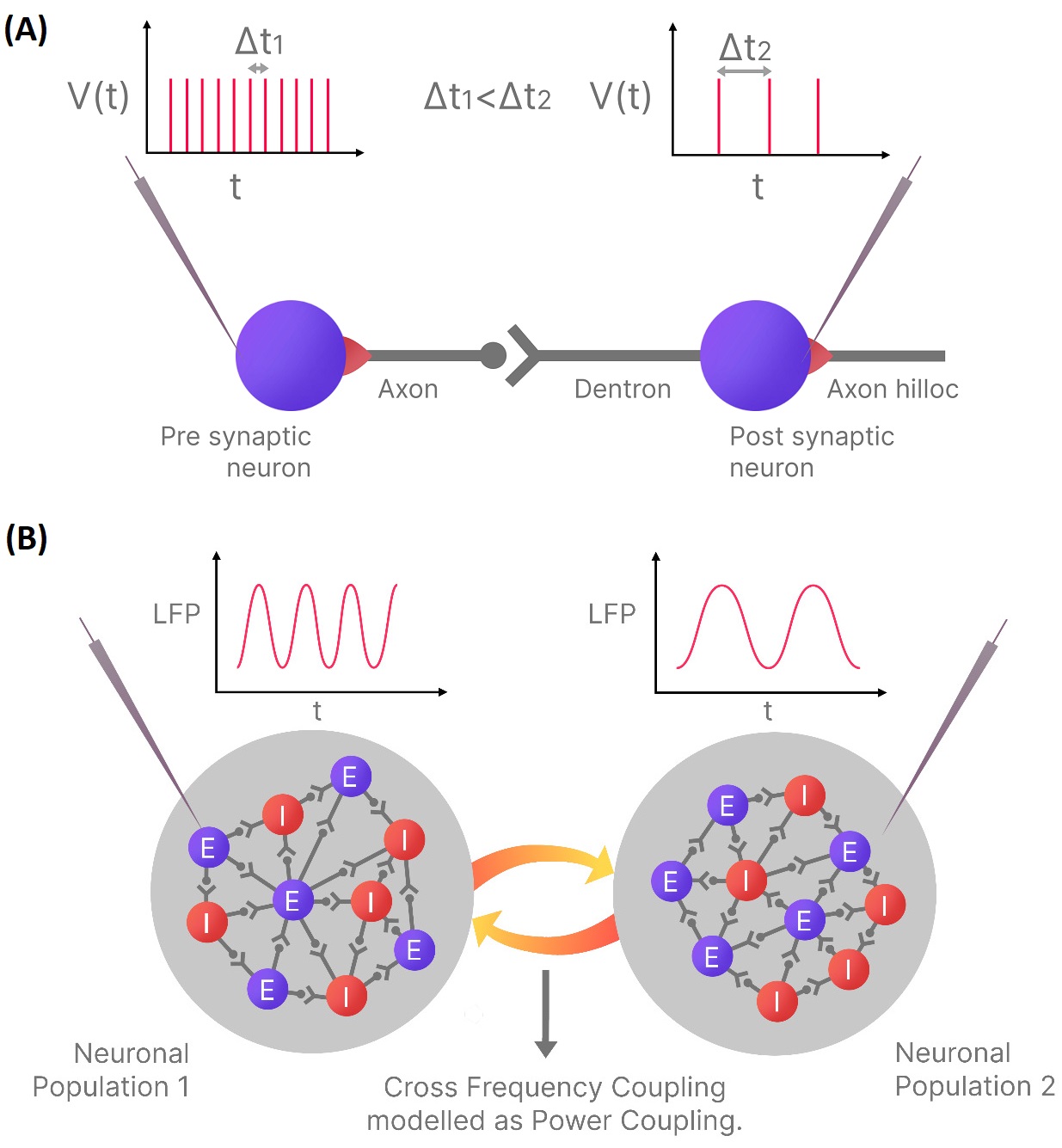

Supplement: Supplementary file 6 [file Image_6.JPEG]
