# Supplementary material for: A Complex-Valued Oscillatory Neural Network for Storage and Retrieval of Multidimensional Aperiodic Signals
Source: Front Comput Neurosci. 2021 May 24;15:551111. doi: 10.3389/fncom.2021.551111 (PMC8181409; doi:10.3389/fncom.2021.551111)
Supplement: Supplementary file 7 [file Table_1.DOCX]

**Supplementary materials:**

**Noise tolerance of the oscillatory network:**

To test the effect of additive noise to the oscillator dynamics on the convergence, a pair of Hopf oscillators with identical natural frequencies coupled through complex coupling is simulated for multiple times for increasing values of noise added to the magnitude of the oscillation as described in the following equation. Where, $N(0, \sigma)$ is the white gaussian noise with zero mean and $\sigma$ standard deviation.

$$\dot{r}_{1}=\left( \mu- r_{1}^{2} \right)r_{1}+Ar_{2}\cos\left( \emptyset_{2}- \emptyset_{1}+ \theta\right)+N(0, \sigma)$$

$$\dot{\emptyset_{1}}=\omega+A\frac{r_{2}}{r_{1}}\sin(\emptyset_{2}-\emptyset_{1}+\theta)$$

$$\dot{r}_{2}=\left( \mu- r_{2}^{2} \right)r_{2}+Ar_{1}\cos\left( \emptyset_{1}- \emptyset_{2}- \theta\right)+N(0, \sigma)$$

$$\dot{\emptyset_{2}}=\omega+A\frac{r_{1}}{r_{2}}\sin(\emptyset_{1}-\emptyset_{2}-\theta)$$

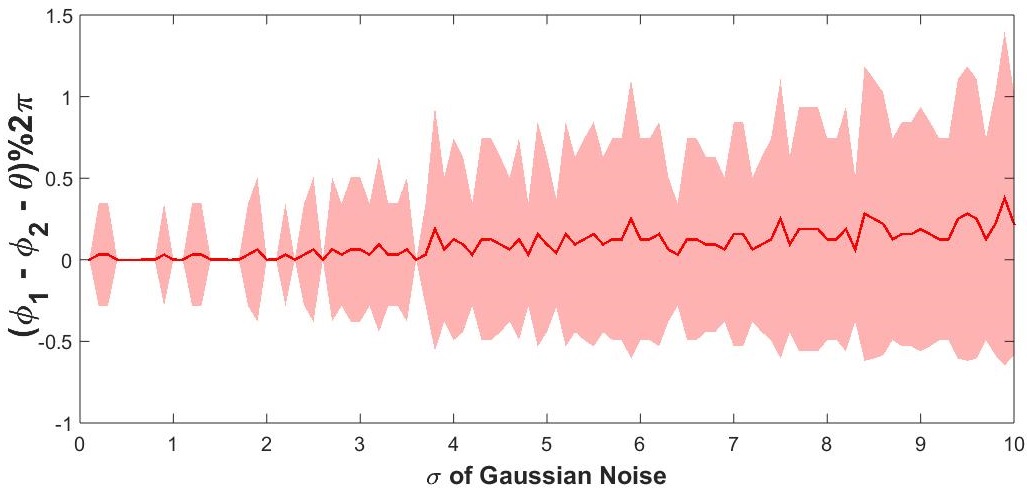


**Fig S1:** In this simulation the following parameters are used: $\omega=5 rad/sec$, $\mu=1$, $\theta=\frac{\pi}{4}$, $A=0.5$. The above-mentioned equations are simulated multiple times for 10 secs to ensure the system achieves steady state, and the mean and the standard deviation value of $\left( \emptyset_{1}-\emptyset_{2}-\theta\right)\%2\pi$ is calculated for increasing values of the standard deviation of gaussian noise. It can be observed that the relative phase difference between the oscillators is still achieving the angle of the complex coupling with reasonable accuracy even though the standard deviation of noise is 10 times larger than the steady state magnitude of oscillation defined by $\mu$.

Next, we verified the effect of additive noise to a pair of Hopf oscillators coupled with tuneable complex coupling weights, driven by noisy complex sinusoidal input signal as described in the following equation. In this case noise is not incorporated in the dynamics of the oscillators.

$$\dot{z}_{1}=z_{1}\left( \mu+i\omega-\left| z_{1} \right|^{2} \right)+Ae^{i\theta}z_{2}+(I_{01}+N(0, \sigma))e^{i(\omega t+\varphi_{1})}$$

$$\dot{z}_{2}=z_{2}\left( \mu+i\omega-\left| z_{2} \right|^{2} \right)+Ae^{-i\theta}z_{1}+(I_{02}+N(0, \sigma))e^{i(\omega t+\varphi_{2})}$$

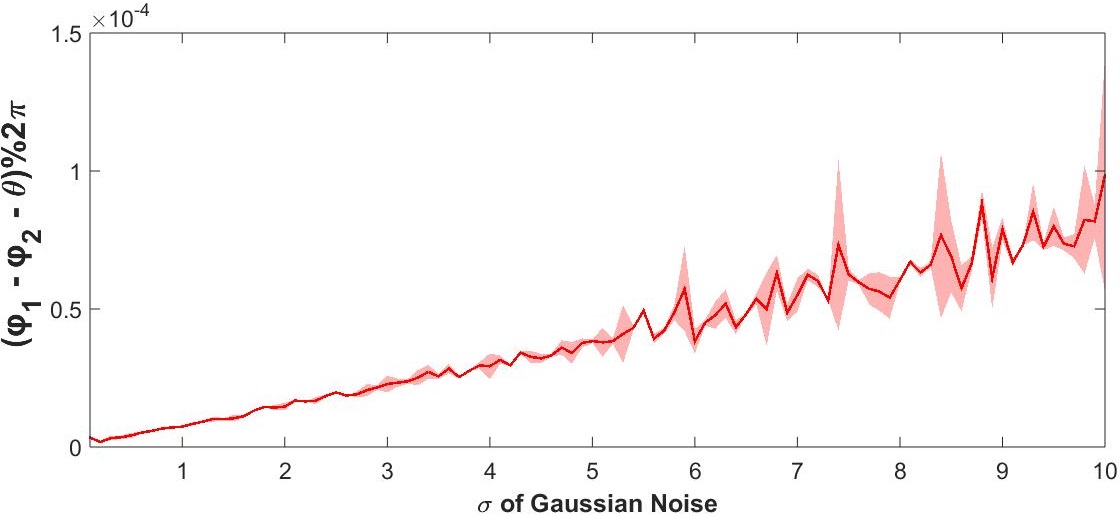


**Fig S2:** The equations mentioned above is simulated multiple times for 10 secs to calculate the mean and the standard deviation of the steady state value of $\left( \varphi_{1}-\varphi_{2}-\theta\right)\%2\pi$ with the increasing values of $\sigma$, for the following set of parameters: $\omega=5 rad/sec$, $\mu=1$, $\varphi_{1}=\frac{\pi}{4}$,$\varphi_{1}=\frac{\pi}{6}$, $A={10}^{-5}$, $I_{01}=I_{02}=0.3$. It can be observed from the plot that there is a linear effect of noise on the steady state value of the angle of the complex coupling and the steady state error between $\varphi_{1}-\varphi_{2}$ and $\theta$ is negligible around the simulated noise margin.

To verify the effect of additive noise in the oscillator dynamics on the dynamics of a pair of Hopf oscillators with nonidentical natural frequencies coupled through power coupling the following equations are simulated with increasing level of noise.

$$\dot{r_{1}}=\left( \mu-{r_{1}}^{2} \right)r_{1}+A_{12}{r_{2}}^{\frac{\omega_{1}}{\omega_{2}}}\cos\omega_{1}\left( \frac{\emptyset_{2}}{\omega_{2}}-\frac{\emptyset_{1}}{\omega_{1}}+\frac{\theta_{12}}{\omega_{1}\omega_{2}} \right)+N(0, \sigma)$$

$$\dot{\emptyset_{1}}=\omega_{1}+A_{12}\frac{{r_{2}}^{\frac{\omega_{1}}{\omega_{2}}}}{r_{1}}\sin\omega_{1}\left( \frac{\emptyset_{2}}{\omega_{2}}-\frac{\emptyset_{1}}{\omega_{1}}+\frac{\theta_{12}}{\omega_{1}\omega_{2}} \right)$$

$$\dot{r_{2}}=\left( \mu-{r_{2}}^{2} \right)r_{2}+A_{21}{r_{1}}^{\frac{\omega_{2}}{\omega_{1}}}\cos\omega_{2}\left( \frac{\emptyset_{1}}{\omega_{1}}-\frac{\emptyset_{2}}{\omega_{2}}+\frac{\theta_{21}}{\omega_{1}\omega_{2}} \right)+N(0, \sigma)$$

$$\dot{\emptyset_{2}}=\omega_{2}+A_{21}\frac{{r_{1}}^{\frac{\omega_{2}}{\omega_{1}}}}{r_{2}}\sin\omega_{2}\left( \frac{\emptyset_{1}}{\omega_{1}}-\frac{\emptyset_{2}}{\omega_{2}}+\frac{\theta_{21}}{\omega_{1}\omega_{2}} \right)$$

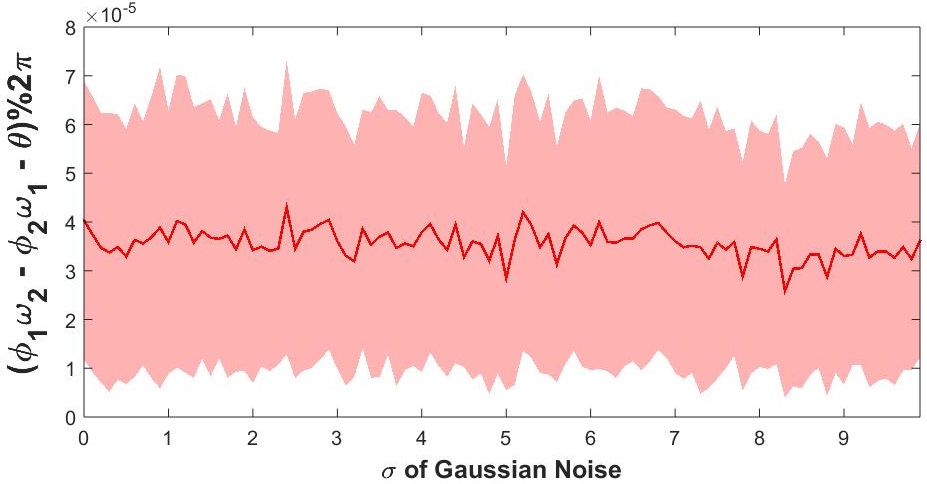


**Fig S3:** The above set of equations are simulated for 40 seconds to find out the gaussian distribution profile of steady state value of $\left( \emptyset_{1}\omega_{2}-\emptyset_{2}\omega_{1}-\theta\right)\%2\pi$ (where $\theta=\theta_{12}$) for the following set of parameters: $\mu=1, \omega_{1}=3,\omega_{2}=4, A=0.5, \theta=\frac{\pi}{4}$. It can be observed that the steady state normalized phase difference $\frac{\emptyset_{1}}{\omega_{1}}-\frac{\emptyset_{2}}{\omega_{2}}$ is nearly independent of the increasing values of noise.

To verify the trainability of power coupling the network presented in section-2.8 is simulated with 3 oscillators with additive noise in the magnitude of the complex input signal: $I_{{ext}_{i}} =(I_{0i}+N\left( 0, \sigma\right))e^{i(w_{i}t + \varphi_{i})}$.


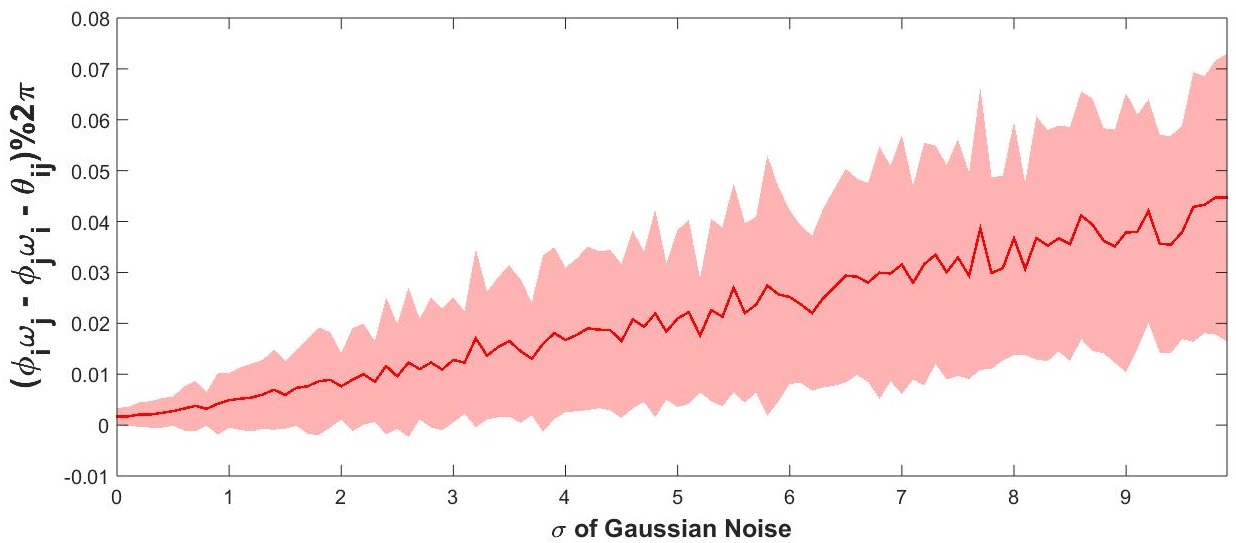


**Fig S4:** Equation-16 is simulated to verify the convergence of $\theta_{ij}$ to $\emptyset_{i}\omega_{j}-\emptyset_{j}\omega_{i}$ or $\varphi_{i}\omega_{j}-\varphi_{j}\omega_{i}$ at steady state for the following set of parameters: $N=3,\omega_{1}=3,\omega_{2}=4,A={10}^{-5}$, $I_{0i}=0.3,\mu=1.$ From the figure it can be concluded that additive noise in the input signal have a linear moderate influence on the entrainment of $\theta_{ij}$.

From the following experiment it is verified that the Fourier decomposition network given in equation-20 is also very robust to the noisy input signal ($D\left( t \right)+N(0,\sigma)$).


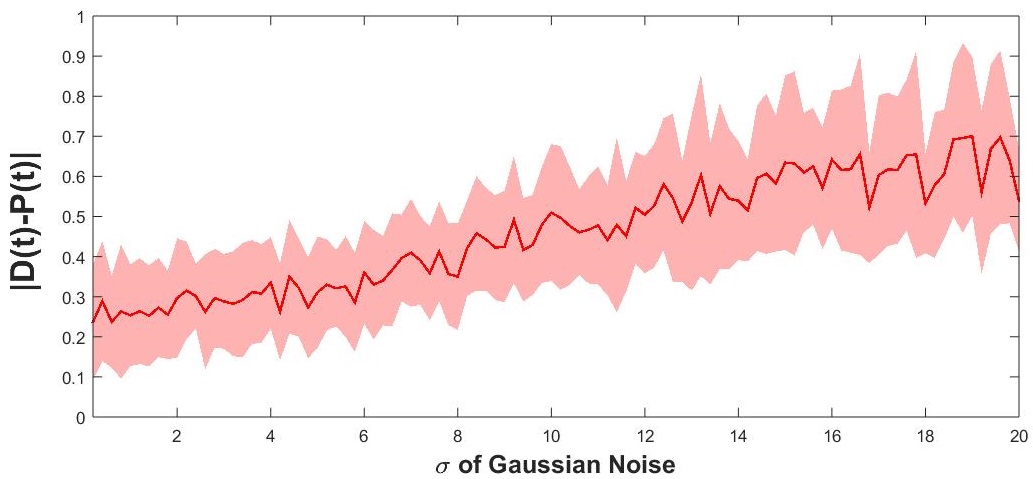


**Fig S5:** The equation-20 is simulated with additive white gaussian noise to the input signal for the following set of parameters: $A_{ij}={10}^{-5},\eta_{\omega}=0.1,\eta_{\alpha}={10}^{-4},\tau_{W}={10}^{4},\varepsilon=0.5, N=3, dt=0.001 sec$. The error plot show that the network is very robust in learning the Fourier decomposition of the input signal.

**Biological plausibility of “power coupling”:**


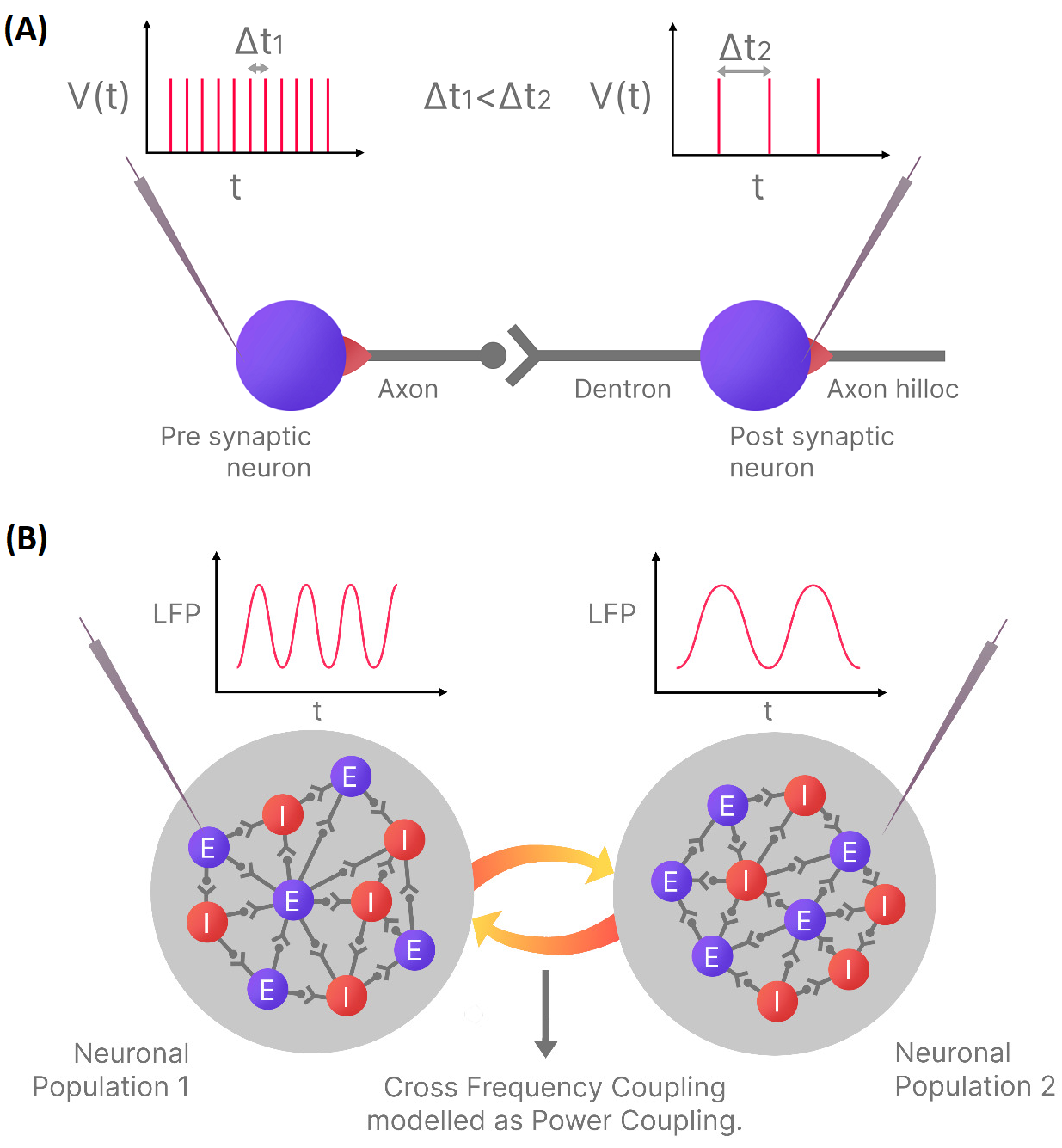


**Fig S6: (A)** At single cell level the electrical properties of the synapse can be such that a presynaptic spike train of a given frequency can be transformed into a post-synaptic spike train of a different frequency and induce power coupling effect on the tonic firing of the presynaptic and the postsynaptic neuron. **(B)** When LFP measurements are taken from clusters of such presynaptic and postsynaptic neurons, assuming both clusters have highly synchronized activity, the coupling between the two clusters can effectively function like “power coupling,” that transforms a presynaptic LFP of a given frequency into a postsynaptic LFP of a different frequency.

**The experimental EEG data:**

The EEG data was open source in the following drive:

<https://drive.google.com/drive/folders/0B0LQHOLfcq-hMFlhOEFKUXFPTkE>

The data was published by:

Grandchamp, R., Braboszcz, C., and Delorme, A. (2014). Oculometric variations during mind wandering. *Front. Psychol.* 5, 1–10. doi:10.3389/fpsyg.2014.00031.

This article is cited by the manuscript.
