# Supplementary material for: A Complex-Valued Oscillatory Neural Network for Storage and Retrieval of Multidimensional Aperiodic Signals
Source: Front Comput Neurosci. 2021 May 24;15:551111. doi: 10.3389/fncom.2021.551111 (PMC8181409; doi:10.3389/fncom.2021.551111)
Supplement: Supplementary file 8 [file Data_Sheet_1.docx]

# Appendix:

**A1. A pair of coupled Hopf oscillators with real coupling:**

The dynamics of a pair of Hopf oscillators represented in equation-2 is given below:

$$\dot{z_{1}}=z_{1}\left( \mu+i\omega_{1}-\left| z_{1} \right|^{2} \right)+W_{21}real\left( z_{2} \right)$$

$$\dot{z_{2}}=z_{2}\left( \mu+i\omega_{2}-\left| z_{2} \right|^{2} \right)+W_{12}real\left( z_{1} \right)$$

Assuming $\omega_{1}=\omega_{2}=\omega$, and $W_{12}$ and $W_{21}$ are real and small, the polar coordinate representation is:

$$\dot{r_{1}}=\left( \mu-{r_{1}}^{2} \right)r_{1}+W_{21}r_{2}\cos\emptyset_{2}\cos\emptyset_{1}$$

$$\dot{\emptyset_{1}}=\omega-W_{21}\frac{r_{2}}{r_{1}}\cos\emptyset_{2}\sin\emptyset_{1}$$

$$\dot{r_{2}}=\left( \mu-{r_{2}}^{2} \right)r_{2}+W_{12}r_{1}\cos\emptyset_{1}\cos\emptyset_{2}$$

$$\dot{\emptyset_{2}}=\omega-W_{12}\frac{r_{1}}{r_{2}}\cos\emptyset_{1}\sin\emptyset_{2}$$

Let the phase difference between the oscillators, $\psi_{12}=\emptyset_{1}-\emptyset_{2}$,

$$\dot{\psi_{12}}=\dot{\emptyset_{1}}-\dot{\emptyset_{2}}$$

$$=W_{12}\frac{r_{1}}{r_{2}}\cos\emptyset_{1}\sin\emptyset_{2}-W_{21}\frac{r_{2}}{r_{1}}\cos\emptyset_{2}\sin\emptyset_{1}$$

At steady state, $\dot{\psi_{12}}=0$, $r_{1}=r_{2}$,

$${\dot{\psi}_{12}}_{ss}=W_{12}\cos\emptyset_{1}\sin\emptyset_{2}-W_{21}\cos\emptyset_{2}\sin\emptyset_{1}=0$$

When $W_{12}=W_{21}=\varepsilon$, a real number,

$${\dot{\psi}_{12}}_{ss}=\varepsilon\left( \cos\emptyset_{1}\sin\emptyset_{2}-\cos\emptyset_{2}\sin\emptyset_{1} \right)=\sin\left( \emptyset_{2}-\emptyset_{1} \right)=0$$

i.e., the solution is: $\emptyset_{2}-\emptyset_{1}=n\pi$*,* $n$ being an integer.

When, $\varepsilon$ is a positive real number, $\emptyset_{2}-\emptyset_{1}$ attains $2n\pi$ solutions, whereas if $\varepsilon$ is negative real number $\emptyset_{2}-\emptyset_{1}$ attains $\left( 2n+1 \right)\pi$ solutions.

**A2. Proof for phase-locking using complex coupling coefficient:**

To ensure two Hopf oscillators with identical natural frequencies be phase-locked at any particular angle independent of the initial condition, the two oscillators need to be coupled using a complex coupling coefficient. With the coupling strategy, as stated in equation-4, it is analytically shown below that the at steady-state two Hopf oscillators will be phase-locked at a particular angle that equals the angle of complex coupling coefficient.

$$\dot{z_{1}}=z_{1}\left( \mu+i\omega_{1}-\left| z_{1} \right|^{2} \right)+Ae^{i\theta}z_{2}$$

$$\dot{z_{2}}=z_{2}\left( \mu+i\omega_{2}-\left| z_{2} \right|^{2} \right)+Ae^{-i\theta}z_{1} \left( a2.1 \right)$$

Let $z_{1}=r_{1}e^{i\emptyset_{1}}$ and $z_{2}=r_{2}e^{i\emptyset_{2}}$, Where $\alpha=\mu(>0),\beta_{1}=-1$, $\omega_{1}=\omega_{2}=\omega$ small $A$;

$$\dot{z_{1}}=\dot{r_{1}}e^{i\emptyset_{1}}+r_{1}e^{i\emptyset_{1}}i\dot{\emptyset_{1}}=e^{i\emptyset_{1}}\left( \dot{r_{1}}+ir_{1}\dot{\emptyset_{1}} \right)$$

$$\dot{z_{2}}=\dot{r_{2}}e^{i\emptyset_{2}}+r_{2}e^{i\emptyset_{2}}i\dot{\emptyset_{2}}=e^{i\emptyset_{2}}\left( \dot{r_{2}}+ir_{2}\dot{\emptyset_{2}} \right) \left( a2.2 \right)$$

Substituting equation-a2.2 in equation-a2.1,

$$e^{i\emptyset_{1}}\left( \dot{r_{1}}+ir_{1}\dot{\emptyset_{1}} \right)=\left( \mu+i\omega_{1}-{r_{1}}^{2} \right)r_{1}e^{i\emptyset_{1}}+Ae^{i\theta}r_{2}e^{i\emptyset_{2}}$$

$$\dot{r_{1}}+ir_{1}\dot{\emptyset_{1}}=\left( \mu+i\omega_{1}-{r_{1}}^{2} \right)r_{1}+Ar_{2}e^{i\left( \emptyset_{2}-\emptyset_{1}+\theta\right)}$$

Similarly,

$$\dot{r_{2}}+ir_{2}\dot{\emptyset_{2}}=\left( \mu+i\omega_{2}-{r_{2}}^{2} \right)r_{2}+Ar_{1}e^{i\left( \emptyset_{1}-\emptyset_{2}-\theta\right)}$$

Equating real and the imaginary term independently,

$$\dot{r_{1}}=\left( \mu-{r_{1}}^{2} \right)r_{1}+Ar_{2}\cos\left( \emptyset_{2}-\emptyset_{1}+\theta\right)$$

$$\dot{\emptyset_{1}}=\omega_{1}+A\frac{r_{2}}{r_{1}}\sin(\emptyset_{2}-\emptyset_{1}+\theta)$$

$$\dot{r_{2}}=\left( \mu-{r_{2}}^{2} \right)r_{2}+Ar_{1}\cos\left( \emptyset_{1}-\emptyset_{2}-\theta\right)$$

$$\dot{\emptyset_{2}}=\omega_{2}+A\frac{r_{1}}{r_{2}}\sin(\emptyset_{1}-\emptyset_{2}-\theta)$$

Let $\psi$ be defined as $\psi=\emptyset_{1}-\emptyset_{2}$

$$\dot{\psi}=\dot{\emptyset_{1}}-\dot{\emptyset_{2}}$$

$$=\omega_{1}-\omega_{2}+A\frac{r_{2}}{r_{1}}\sin\left( -\left( \psi-\theta\right) \right)-A\frac{r_{1}}{r_{2}}\sin(\psi-\theta)$$

$$=\omega_{1}-\omega_{2}-\frac{A{r_{2}}^{2}\sin(\psi-\theta)+A{r_{1}}^{2}\sin(\psi-\theta)}{r_{1}r_{2}}$$

In this scenario $\omega_{1}=\omega_{2}$ and at steady state $r_{1}=r_{2}$, $\dot{\psi_{ss}}=0$,

$$\dot{\psi_{ss}}=2A\sin(\theta-\psi_{ss})=0$$

Implying that,

$$\psi_{ss}-\theta=2n\pi$$

$$\psi_{ss}=2n\pi+\theta$$

$${\emptyset_{1}}_{ss}-{\emptyset_{2}}_{ss}=2n\pi+\theta$$

**A3. Stability of symmetric steady state nonzero solution:**

Referring the dynamics of equation-5a and 5b (Hebbian like learning for complex coupling coefficient):

$$\tau_{W}\dot{A}=-A+r_{1}r_{2}\cos(\emptyset_{1}-\emptyset_{2}-\theta)$$

$$\tau_{W}\dot{\theta}=\frac{r_{1}r_{2}}{A}\sin(\emptyset_{1}-\emptyset_{2}-\theta)$$

As we have limited the scope of our study only to $\theta$ dynamics by assuming $\dot{A}=0,$on the symmetric plane ($r_{1}=r_{2}, \sigma_{12}=-\sigma_{21}=\sigma=\theta-\emptyset_{1}+\emptyset_{2}$) the dynamics above with the dynamics in equation-4 stands:

$$\dot{r}=\mu r-r^{3}+Ar\cos\sigma$$

$$\dot{\sigma}=-\left( 2A+\frac{r^{2}}{\tau_{W}A} \right)\sin\sigma(a3.1)$$

This system has different steady state solutions. The solutions are:

$$r^{*}=\sqrt{\mu+A}, 0; \sigma^{*}=n\pi$$

It can be observed that for $A\ll\mu$, $r^{*}\cong\sqrt{\mu}$.

The Jacobian matrix of the dynamical system of equation-a3.1:

$$J=\left[ \begin{matrix} \frac{\partial f}{\partial r} & \frac{\partial f}{\partial\sigma} \\ \frac{\partial g}{\partial r} & \frac{\partial g}{\partial\sigma} \end{matrix} \right]=\left[ \begin{matrix} \mu-3r^{2}+A\cos\sigma& -Ar\sin\sigma\\ -\frac{2r}{\tau_{W}A}\sin\sigma& -\left( 2A+\frac{r^{2}}{\tau_{W}A} \right)\cos\sigma\end{matrix} \right]$$

The Jacobian matrix at the solution: $r^{*}=\sqrt{\mu+A}, \sigma^{*}=n\pi$.

$$J=\left[ \begin{matrix} -2\left( \mu+A \right) & 0 \\ 0 & -\left( 2A+\frac{\mu+A}{\tau_{W}A} \right) \end{matrix} \right]$$

It can be observed that both the eigen values are negative. So, the nonzero symmetric solution is a stable node for $A\ll\mu$. Whereas the Jacobian matrix at $r^{*}=0, \sigma^{*}=n\pi$

$$J=\left[ \begin{matrix} \mu+A & 0 \\ 0 & -2A \end{matrix} \right]$$

So, for $r^{*}=0, \sigma^{*}=n\pi$, one of the eigen value is positive whereas the other is negative, implying that the zero solution is a saddle node.

**A4. Two complex Hopf oscillators with power coupling:**

Consider two supercritical Hopf oscillators with different natural frequencies coupled as described in the following equations. We explore under the steady-state condition, what would be the phase relationship of the two oscillators.

$$\dot{z_{1}}=z_{1}\left( \mu+i\omega_{1}-\left| z_{1} \right|^{2} \right)+Ae^{i\frac{\theta}{\omega_{2}}}{(z_{2})}^{\frac{\omega_{1}}{\omega_{2}}}$$

$$\dot{z_{2}}=z_{2}\left( \mu+i\omega_{2}-\left| z_{2} \right|^{2} \right)+Ae^{-i\frac{\theta}{\omega_{1}}}{(z_{1})}^{\frac{\omega_{2}}{\omega_{1}}} (a4.1)$$

Let $z_{1}=r_{1}e^{i\emptyset_{1}}$ and $z_{2}=r_{2}e^{i\emptyset_{2}}$, where $\alpha=\mu(>0),\beta_{1}=-1$,

$$\dot{z_{1}}=\dot{r_{1}}e^{i\emptyset_{1}}+r_{1}e^{i\emptyset_{1}}i\dot{\emptyset_{1}}=e^{i\emptyset_{1}}\left( \dot{r_{1}}+ir_{1}\dot{\emptyset_{1}} \right)$$

$$\dot{z_{2}}=\dot{r_{2}}e^{i\emptyset_{2}}+r_{2}e^{i\emptyset_{2}}i\dot{\emptyset_{2}}=e^{i\emptyset_{2}}\left( \dot{r_{2}}+ir_{2}\dot{\emptyset_{2}} \right) \left( a4.2 \right)$$

Substituting equation-a4.2 into equation-a4.1,

$$e^{i\emptyset_{1}}\left( \dot{r_{1}}+ir_{1}\dot{\emptyset_{1}} \right)=\left( \mu+i\omega_{1}-{r_{1}}^{2} \right)r_{1}e^{i\emptyset_{1}}+Ae^{i\frac{\theta}{\omega_{2}}}{(r_{2})}^{\frac{\omega_{1}}{\omega_{2}}}e^{i\emptyset_{2}\frac{\omega_{1}}{\omega_{2}}}$$

$$\dot{r_{1}}+ir_{1}\dot{\emptyset_{1}}=\left( \mu+i\omega_{1}-{r_{1}}^{2} \right)r_{1}+A{(r_{2})}^{\frac{\omega_{1}}{\omega_{2}}}e^{i\left( \emptyset_{2}\frac{\omega_{1}}{\omega_{2}}-\emptyset_{1}+\frac{\theta}{\omega_{2}} \right)}$$

$$=\left( \mu+i\omega_{1}-{r_{1}}^{2} \right)r_{1}+A{(r_{2})}^{\frac{\omega_{1}}{\omega_{2}}}e^{i\omega_{1}\left( \frac{\emptyset_{2}}{\omega_{2}}-\frac{\emptyset_{1}}{\omega_{1}}+\frac{\theta}{\omega_{1}\omega_{2}} \right)}$$

Similarly,

$$\dot{r_{2}}+ir_{2}\dot{\emptyset_{2}}=\left( \mu+i\omega_{2}-{r_{2}}^{2} \right)r_{2}+A{(r_{1})}^{\frac{\omega_{2}}{\omega_{1}}}e^{i\omega_{2}\left( \frac{\emptyset_{1}}{\omega_{1}}-\frac{\emptyset_{2}}{\omega_{2}}-\frac{\theta}{\omega_{1}\omega_{2}} \right)}$$

Equating the real and imaginary terms,

$$\dot{r_{1}}=\left( \mu-{r_{1}}^{2} \right)r_{1}+A{(r_{2})}^{\frac{\omega_{1}}{\omega_{2}}}\cos\omega_{1}\left( \frac{\emptyset_{2}}{\omega_{2}}-\frac{\emptyset_{1}}{\omega_{1}}+\frac{\theta}{\omega_{1}\omega_{2}} \right)$$

$$\dot{\emptyset_{1}}=\omega_{1}+A\frac{{(r_{2})}^{\frac{\omega_{1}}{\omega_{2}}}}{r_{1}}\sin\omega_{1}\left( \frac{\emptyset_{2}}{\omega_{2}}-\frac{\emptyset_{1}}{\omega_{1}}+\frac{\theta}{\omega_{1}\omega_{2}} \right)$$

$$\dot{r_{2}}=\left( \mu-{r_{2}}^{2} \right)r_{2}+A{(r_{1})}^{\frac{\omega_{2}}{\omega_{1}}}\cos\omega_{2}\left( \frac{\emptyset_{1}}{\omega_{1}}-\frac{\emptyset_{2}}{\omega_{2}}-\frac{\theta}{\omega_{1}\omega_{2}} \right)$$

$$\dot{\emptyset_{2}}=\omega_{2}+A\frac{{(r_{1})}^{\frac{\omega_{2}}{\omega_{1}}}}{r_{2}}\sin\omega_{2}\left( \frac{\emptyset_{1}}{\omega_{1}}-\frac{\emptyset_{2}}{\omega_{2}}-\frac{\theta}{\omega_{1}\omega_{2}} \right)$$

At steady-state, the relationship between two oscillator's natural frequencies $\omega_{1}$ and $\omega_{2}$ and phase $\emptyset_{1}$ and $\emptyset_{2}$ respectively holds the following relation,

$$\frac{{\emptyset_{1}}_{ss}}{\omega_{1}}-\frac{{\emptyset_{2}}_{ss}}{\omega_{2}}=constant$$

$${\emptyset_{1}}_{ss}\omega_{2}-{\emptyset_{2}}_{ss}\omega_{1}=constant$$

This constant can be any real number. Let’s denote $\psi$ as $\frac{\emptyset_{1}}{\omega_{1}}-\frac{\emptyset_{2}}{\omega_{2}}$, as we know that $\psi$ is constant at steady state, hence $\dot{\psi_{ss}}=0$

$$\dot{\psi_{ss}}=\frac{\dot{{\emptyset_{1}}_{ss}}}{\omega_{1}}-\frac{\dot{{\emptyset_{2}}_{ss}}}{\omega_{2}}$$

$$=\frac{A}{\omega_{1}}\frac{{r_{2}}^{\frac{\omega_{1}}{\omega_{2}}}}{r_{1}}\sin\omega_{1}\left( -\psi_{ss}+\frac{\theta}{\omega_{1}\omega_{2}} \right)-\frac{A}{\omega_{2}}\frac{{(r_{1})}^{\frac{\omega_{2}}{\omega_{1}}}}{r_{2}}\sin\omega_{2}\left( \psi_{ss}-\frac{\theta}{\omega_{1}\omega_{2}} \right)$$

$$=\frac{A}{\omega_{1}}\frac{{r_{2}}^{\frac{\omega_{1}}{\omega_{2}}}}{r_{1}}\sin\omega_{1}\left( \frac{\theta}{\omega_{1}\omega_{2}}-\psi_{ss} \right)+\frac{A}{\omega_{2}}\frac{{(r_{1})}^{\frac{\omega_{2}}{\omega_{1}}}}{r_{2}}\sin\omega_{2}\left( \frac{\theta}{\omega_{1}\omega_{2}}-\psi_{ss} \right)$$

At steady-state $r_{1}\cong r_{2}\cong1$ as $\mu$ for both of the oscillators equals 1 and $A\ll1$, the previous equation becomes,

$$\dot{\psi_{ss}}=\frac{A}{\omega_{1}}\sin\omega_{1}\left( \frac{\theta}{\omega_{1}\omega_{2}}-\psi_{ss} \right)+\frac{A}{\omega_{2}}\sin\omega_{2}\left( \frac{\theta}{\omega_{1}\omega_{2}}-\psi_{ss} \right)$$

As $\dot{\psi_{ss}}=0$, implying that, for a subset (say $ѡ$) of solutions:

$$\omega_{1}\left( \frac{\theta}{\omega_{1}\omega_{2}}-\psi_{ss} \right)=\pm n_{1}\pi$$

$$\psi_{ss}-\frac{\theta}{\omega_{1}\omega_{2}}=\pm\frac{n_{1}\pi}{\omega_{1}}$$

$$\psi_{ss}=\frac{\theta}{\omega_{1}\omega_{2}}\pm\frac{n_{1}\pi}{\omega_{1}} (a4.3)$$

as well as,

$$\omega_{2}\left( \frac{\theta}{\omega_{1}\omega_{2}}-\psi_{ss} \right)=n_{2}\pi$$

$$\psi_{ss}=\frac{\theta}{\omega_{1}\omega_{2}}\pm\frac{n_{2}\pi}{\omega_{2}} (a4.4)$$

From the above equation-a4.3 and a4.4, when $n_{1}=n_{1}\neq0$ (other than the desired solution for which $n_{1}=n_{1}=0$), the subset $ѡ$ will only contain desired solution when $\frac{\omega_{1}}{\omega_{2}}$ is an irrational number.

$$\frac{n_{1}}{n_{2}}=\frac{\omega_{1}}{\omega_{2}}$$

**A5. Derivation of learning rule for power coupling weight:**

If $N$ of supercritical Hopf oscillators is laterally connected with each other through power coupling, the dynamical equations will look like:

$$\dot{z_{i}}=\left( \mu+i\omega_{i}-\left| z_{i} \right|^{2} \right)z_{i}+\sum_{j} A_{ij}e^{i\frac{\theta_{ij}}{\omega_{j}}}{z_{j}}^{\frac{\omega_{i}}{\omega_{j}}} (a5.1)$$

where $z_{i}=r_{i}e^{i\emptyset_{i}}$

Therefore,

$$\dot{z_{i}}=\dot{r_{i}}e^{i\emptyset_{i}}+ir_{i}\dot{\emptyset_{i}}e^{i\emptyset_{i}}=\left( \mu-{r_{i}}^{2} \right)r_{i}e^{i\emptyset_{i}}+i\omega_{i}r_{i}e^{i\emptyset_{i}}+\sum_{j} A_{ij}e^{i\frac{\theta_{ij}}{\omega_{j}}}{r_{j}}^{\frac{\omega_{i}}{\omega_{j}}}e^{i\emptyset_{j}\frac{\omega_{i}}{\omega_{j}}}$$

$$\dot{r_{i}}+ir_{i}\dot{\emptyset_{i}}=\left( \mu-{r_{i}}^{2} \right)r_{i}+i\omega_{i}r_{i}+\sum_{j} A_{ij}{r_{j}}^{\frac{\omega_{i}}{\omega_{j}}}e^{i\omega_{i}\left( \frac{\emptyset_{j}}{\omega_{j}}-\frac{\emptyset_{i}}{\omega_{i}}+\frac{\theta_{ij}}{\omega_{i}\omega_{j}} \right)}$$

Separating real and imaginary part;

$$\dot{r_{i}}=\left( \mu-{r_{i}}^{2} \right)r_{i}+\sum_{j} A_{ij}{r_{j}}^{\frac{\omega_{i}}{\omega_{j}}}\cos\omega_{i}\left( \frac{\emptyset_{j}}{\omega_{j}}-\frac{\emptyset_{i}}{\omega_{i}}+\frac{\theta_{ij}}{\omega_{i}\omega_{j}} \right) (a5.2)$$

$$\dot{\emptyset_{i}}=\omega_{i}+\sum_{j} A_{ij}\frac{{r_{j}}^{\frac{\omega_{i}}{\omega_{j}}}}{r_{i}}\sin\omega_{i}\left( \frac{\emptyset_{j}}{\omega_{j}}-\frac{\emptyset_{i}}{\omega_{i}}+\frac{\theta_{ij}}{\omega_{i}\omega_{j}} \right) (a5.3)$$

The normalized phase difference between $i^{th}$ and $j^{th}$ Hopf oscillator,

$$\psi_{ij}=\frac{\emptyset_{i}}{\omega_{i}}-\frac{\emptyset_{j}}{\omega_{j}} (a5.4)$$

Therefore, from equation-a5.3 and a5.4,

$$\dot{\psi_{ij}}=\frac{\dot{\emptyset_{i}}}{\omega_{i}}-\frac{\dot{\emptyset_{j}}}{\omega_{j}}=\frac{\omega_{i}+\sum_{k} A_{ik}\frac{{r_{k}}^{\frac{\omega_{i}}{\omega_{k}}}}{r_{i}}\sin\omega_{i}\left( \frac{\emptyset_{k}}{\omega_{k}}-\frac{\emptyset_{i}}{\omega_{i}}+\frac{\theta_{ik}}{\omega_{i}\omega_{k}} \right)}{\omega_{i}}-\frac{\omega_{j}+\sum_{k} A_{jk}\frac{{r_{k}}^{\frac{\omega_{j}}{\omega_{k}}}}{r_{j}}\sin\omega_{j}\left( \frac{\emptyset_{k}}{\omega_{k}}-\frac{\emptyset_{j}}{\omega_{j}}+\frac{\theta_{jk}}{\omega_{j}\omega_{k}} \right)}{\omega_{j}}$$

Or, $\dot{\psi_{ij}}=\sum_{k} A_{ik}\frac{{r_{k}}^{\frac{\omega_{i}}{\omega_{k}}}}{r_{i}}\sin\omega_{i}\left( \frac{\emptyset_{k}}{\omega_{k}}-\frac{\emptyset_{i}}{\omega_{i}}+\frac{\theta_{ik}}{\omega_{i}\omega_{k}} \right)-\sum_{k} A_{jk}\frac{{r_{k}}^{\frac{\omega_{j}}{\omega_{k}}}}{r_{j}}\sin\omega_{j}\left( \frac{\emptyset_{k}}{\omega_{k}}-\frac{\emptyset_{j}}{\omega_{j}}+\frac{\theta_{jk}}{\omega_{j}\omega_{k}} \right) (a5.5)$

At steady state, $\dot{\psi_{ij}}=0$

and, ${Z_{i}}_{ss}\left( t \right)={r_{i}}_{ss}e^{i{\theta_{i}\left( t \right)}_{ss}}={r_{i}}_{ss}e^{i(\omega_{i}t+{\beta_{i}}_{ss})}$

where ${\beta_{i}}_{ss}$ and ${r_{i}}_{ss}$ are the steady state phase offset and magnitude of the $i^{th}$ oscillator.

From equation-a5.5,

$$\sum_{k} A_{ik}\frac{{{r_{k}}_{ss}}^{\frac{\omega_{i}}{\omega_{k}}}}{{r_{i}}_{ss}}\sin\omega_{i}\left( \frac{{\theta_{k}}_{ss}}{\omega_{k}}-\frac{{\theta_{i}}_{ss}}{\omega_{i}}+\frac{\emptyset_{ik}}{\omega_{i}\omega_{k}} \right)-\sum_{k} A_{jk}\frac{{{r_{k}}_{ss}}^{\frac{\omega_{j}}{\omega_{k}}}}{{r_{j}}_{ss}}\sin\omega_{i}\left( \frac{{\theta_{k}}_{ss}}{\omega_{k}}-\frac{{\theta_{j}}_{ss}}{\omega_{j}}+\frac{\emptyset_{jk}}{\omega_{j}\omega_{k}} \right)=0 (a5.6)$$

One obvious of equation-a5.6 is,

$$\frac{{\theta_{i}}_{ss}}{\omega_{i}}-\frac{{\theta_{j}}_{ss}}{\omega_{j}}=\frac{\emptyset_{ij}}{\omega_{i}\omega_{j}}$$

$${\theta_{i}}_{ss}\omega_{j}-{\theta_{j}}_{ss}\omega_{i}=\emptyset_{ij} (a5.7)$$

Thus, at the time of learning the Fourier decomposition of a given perturbing input signal, to restore the normalized phase of oscillations of the Hopf oscillators $\emptyset_{ij}$, the angle of complex lateral coupling has to be updated according to equation-a4.7, which boils down to the following learning rule of the lateral connection:

$$\Delta W_{ij}=-A_{ij}e^{i\frac{\emptyset_{ij}}{\omega_{j}}}+\left( {r_{i}}^{\omega_{j}}{r_{j}}^{\omega_{i}}e^{i\left( \theta_{i}\omega_{j}-\theta_{j}\omega_{i} \right)} \right)^{\frac{1}{\omega_{j}}}$$

$$\Delta W_{ij}=-A_{ij}e^{i\frac{\emptyset_{ij}}{\omega_{j}}}+r_{i}{r_{j}}^{\frac{\omega_{i}}{\omega_{j}}}e^{i\frac{\left( \theta_{i}\omega_{j}-\theta_{j}\omega_{i} \right)}{\omega_{j}}}$$

$$\Delta W_{ij}=-A_{ij}e^{i\frac{\emptyset_{ij}}{\omega_{j}}}+r_{i}{r_{j}}^{\frac{\omega_{i}}{\omega_{j}}}e^{i\left( \theta_{i}-\frac{\theta_{j}\omega_{i}}{\omega_{j}} \right)}$$

$$\Delta W_{ij}=-W_{ij}+Z_{i}\left( {Z_{j}}^{*} \right)^{\frac{\omega_{i}}{\omega_{j}}} (a5.8)$$

**A6. Derivation of the complex feed-forward weights in the generative network:**

For the batch mode of learning the squared loss function is as defined;

$$L=\frac{1}{2}\sum_{i=1}^{M} \sum_{t} \left\| {Y_{d}}_{i}\left( t \right)-{Y_{p}}_{i}\left( t \right) \right\|^{2} (a6.1)$$

where,

$${Y_{p}}_{i}\left( t \right)= real\left( \sum_{j=1}^{n} W_{ij}e^{i\emptyset_{j}} \right) (a6.2)$$

and $W_{ij}$ is the complex output weight from $j^{th}$ Kuramoto oscillator to the $i^{th}$ output node, defined as;

$$W_{ij}= K_{ij}e^{i\zeta_{ij}}$$

From equation-a6.1,

$${Y_{p}}_{i}\left( t \right)= real\left( \sum_{j=1}^{n} K_{ij}e^{i\left( \theta_{j}+\zeta_{ij} \right)} \right)=\sum_{j=1}^{n} K_{ij}\cos\left( \theta_{j}+\zeta_{ij} \right) (a6.3)$$

From equation-a6.1 and a6.3 the update rule for $K_{ij}$ and $\emptyset_{ij}$ can be derived as;

$$\Delta K_{ij}=\eta_{K}\frac{\partial L}{\partial K_{ij}}=\frac{1}{2}\eta_{K}\frac{\partial}{\partial K_{ij}}\left( \sum_{t} \left\| {Y_{d}}_{i}\left( t \right)-{Y_{p}}_{i}\left( t \right) \right\|^{2} \right)=\left( -1 \right)\eta_{K}\sum_{t} \left( {Y_{d}}_{i}\left( t \right)-{Y_{p}}_{i}\left( t \right) \right)\frac{\partial{Y_{p}}_{i}\left( t \right)}{\partial K_{ij}}$$

or, $\Delta K_{ij}=\left( -1 \right)\eta_{K}\sum_{t} \left( {Y_{d}}_{i}\left( t \right)-{Y_{p}}_{i}\left( t \right) \right)\frac{\partial}{\partial K_{ij}}\left( \sum_{k=1}^{n} K_{ik}\cos\left( \emptyset_{k}+\zeta_{ik} \right) \right)$

or, $\Delta K_{ij}=\left( -1 \right)\eta_{K}\sum_{t} \left( {Y_{d}}_{i}\left( t \right)-{Y_{p}}_{i}\left( t \right) \right)\cos\left( \emptyset_{j}+\zeta_{ij} \right) (a6.4)$

Similarly;

$$\Delta\zeta_{ij}=\eta_{\zeta}\frac{\partial L}{\partial\zeta_{ij}}=\frac{1}{2}\eta_{\zeta}\frac{\partial}{\partial\zeta_{ij}}\left( \sum_{t} \left\| {Y_{d}}_{i}\left( t \right)-{Y_{p}}_{i}\left( t \right) \right\|^{2} \right)=\left( -1 \right)\eta_{\zeta}\sum_{t} \left( {Y_{d}}_{i}\left( t \right)-{Y_{p}}_{i}\left( t \right) \right)\frac{\partial{Y_{p}}_{i}\left( t \right)}{\partial\zeta_{ij}}$$

or, $\Delta\zeta_{ij}=\left( -1 \right)\eta_{\zeta}\sum_{t} \left( {Y_{d}}_{i}\left( t \right)-{Y_{p}}_{i}\left( t \right) \right)\frac{\partial}{\partial\zeta_{ij}}\left( \sum_{k=1}^{n} K_{ik}\cos\left( \emptyset_{k}+\zeta_{ik} \right) \right)$

or, $\Delta\zeta_{ij}=\left( -1 \right)\eta_{\zeta}\sum_{t} \left( {Y_{d}}_{i}\left( t \right)-{Y_{p}}_{i}\left( t \right) \right)\left( -K_{ij}\sin\left( \emptyset_{j}+\zeta_{ij} \right) \right) (a6.5)$
